# POxload: Machine Learning Estimates Drug Loadings of Polymeric Micelles

**DOI:** 10.1021/acs.molpharmaceut.4c00086

**Published:** 2024-05-28

**Authors:** Josef Kehrein, Alex Bunker, Robert Luxenhofer

**Affiliations:** † Soft Matter Chemistry, Department of Chemistry, Faculty of Science, University of Helsinki, A. I. Virtasen aukio 1, 00014 Helsinki, Finland; ‡ Drug Research Program, Division of Pharmaceutical Biosciences Faculty of Pharmacy, University of Helsinki, Viikinkaari 5 E, 00014 Helsinki, Finland

**Keywords:** drug delivery, hydrophobic drugs, poly(2-oxazoline), poly(2-oxazine), polymer micelles, solubility, quantitative structure–property relationship

## Abstract

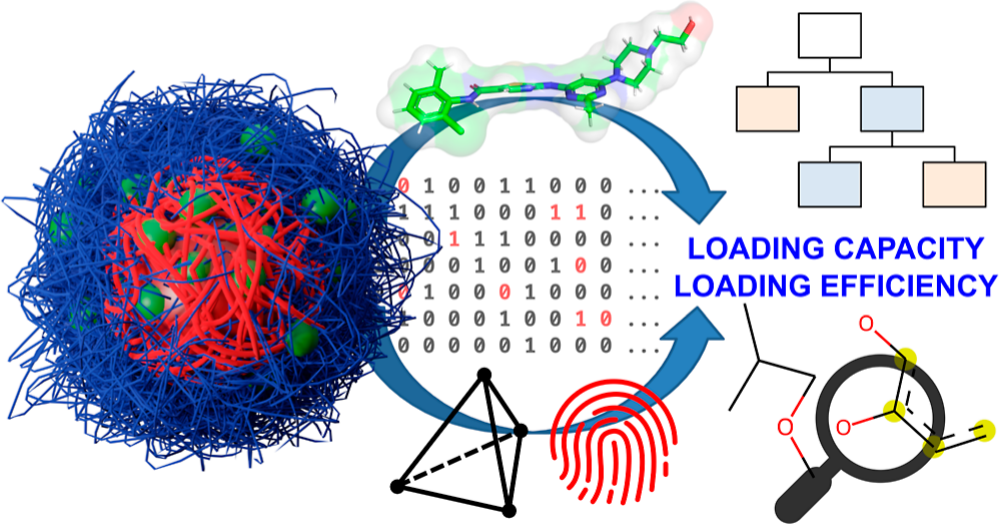

Block copolymers, composed of poly(2-oxazoline)s and poly(2-oxazine)s, can serve as drug delivery systems; they form micelles that carry poorly water-soluble drugs. Many recent studies have investigated the effects of structural changes of the polymer and the hydrophobic cargo on drug loading. In this work, we combine these data to establish an extended formulation database. Different molecular properties and fingerprints are tested for their applicability to serve as formulation-specific mixture descriptors. A variety of classification and regression models are built for different descriptor subsets and thresholds of loading efficiency and loading capacity, with the best models achieving overall good statistics for both cross- and external validation (balanced accuracies of 0.8). Subsequently, important features are dissected for interpretation, and the DrugBank is screened for potential therapeutic use cases where these polymers could be used to develop novel formulations of hydrophobic drugs. The most promising models are provided as an open-source software tool for other researchers to test the applicability of these delivery systems for potential new drug candidates.

## Introduction

Poor solubility of therapeutic substances imposes an increasing challenge on the pharmaceutical industry. Around 40% of approved drugs and even more candidates within the pipeline suffer from this pharmakokinetic obstacle.^1,2^ This has shifted the focus of scientists to the development of novel drug delivery systems (DDS) that function as carriers to efficiently transport hydrophobic drugs to their therapeutic target.^3^*Prima facie*, the ever-increasing chemical diversity of sophisticated nanotechnologies theoretically available for drug formulation should provide researchers with a suitable arsenal to overcome most solubility issues. In practice, due to the lack of mechanistic insight into the driving forces within such DDS, formulation development is still driven mainly heuristically by time- and resource-intensive experimental screenings; this has resulted in only very limited cases of alternative delivery systems to be used in marketed therapeutics.^4^

It is therefore of great interest for the pharmaceutical research community to optimize development processes *via* the integration of complementary *in silico* methods. High-throughput virtual screenings employing techniques like molecular docking are already well-established during the phase of early drug design.^5^ In recent years, increasing amounts of computational resources and available experimental data have also allowed molecular dynamics simulations,^4^ machine learning,^6^ or quantitative structure–property relationship (QSPR) modeling^7^ to guide researchers during the later stage of drug formulation. Thus, the term “computational pharmaceutics” has emerged to describe the usage of *in silico* methods for the purpose of optimizing pharmaceutical technologies and drug delivery processes.^8^ While QSPR modeling is traditionally performed by correlation of small molecule descriptors to physicochemical or pharmacological properties,^7^ its usage has expanded to other fields^9^ including studies on mixtures of compounds,^10−12^ predicting chemical reactions,^13,14^ or characterization of various polymer properties.^15−19^ In this regard, “polymer informatics”^20^ represents a rather new but active field of research and various types of representations of macromolecular structures for QSPR modeling and machine learning pipelines have been described, including (Big)SMILES strings,^21,22^ classical descriptors, fingerprints and binary images,^23^ or, more recently, methods inspired by natural language processing.^24,25^

Previous QSPR modeling approaches with respect to polymers have focused on determining various properties important to the materials science community,^17^ including, among others, dielectric constants,^26^ refractive indices,^27,28^ glass transition temperatures,^29,30^ viscosity,^31^ or fouling release activity.^32^ Although the chemical nature of macromolecular structures usually prevents direct applications of classical molecular descriptor generation methods to whole polymeric systems, the referenced studies include many examples of classification and regression models with high predictive performance. In these instances, this was achieved by representation of the respective polymers by their much smaller monomer repeating units, usually capped with hydrogens, that enable researchers to compute quantum-chemical or classical 2D/3D molecular descriptors. While these studies illustrate the applicability of such descriptors, as mentioned above, the development of optimal polymer modeling approaches for QSPR studies is an ongoing endeavor. As polymers represent a broad class of substances relevant to many research fields, this inevitably raises questions on how to best model mixtures of various components, including copolymers,^27,28^ as well as the presence of different solvents^33,34^ or, within pharmaceutical sciences, drug molecules.^35,36^

**Figure 1:**
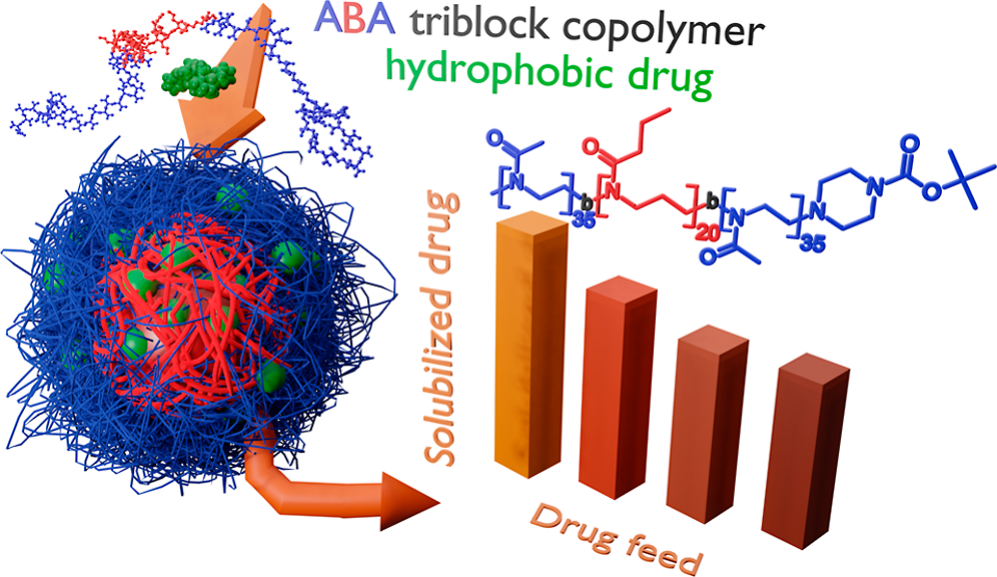
Schematic drawing of a polymeric micelle^45^ consisting of amphiphilic ABA-triblock copolymers. Outer hydrophilic A blocks are shown in blue, B blocks in red, and hydrophobic guest molecules in green. The chemical structure of a polymer consisting of pMeOx A and pPrOzi B blocks (A-nPrOzi-A) is illustrated on the right. The amount of solubilized drug can be characterized at different DF with a constant polymer feed (usually 10 g/L), which provides information on LC and LE.

Polymeric micelles represent one of the many pharmaceutical delivery strategies that has garnered much attention but limited translation.^37,38^ For example, within the past decade amphiphilic ABA-triblock copolymers consisting of poly(2-oxazoline) (pOx) and poly(2-oxazine) (pOzi) with readily tunable sidechains showed great value as carrier for hydrophobic drugs with therapeutic value, *e.g.,* anticancer agents like paclitaxel.^39−41^ In the presence of drugs, these polymers can form micelles where the hydrophilic A blocks form a protective “corona” around the inner hydrophobic B blocks as the main drug carrier (Figure 1). However, this image is too simplistic as A blocks have shown to also interact with drugs, questioning the widely established but rather simplistic picture of a core–shell architecture.^42−48^ Poly(2-methyl-2-oxazoline) (pMeOx) is usually used as A blocks, whereas for B block sidechains of a length of three to four carbon atoms, including poly(2-butyl-2-oxazoline) (pBuOx), poly(2-butyl-2-oxazine) (pBuOzi), poly(2-propyl-2-oxazoline) (pPrOx), and poly(2-propyl-2-oxazine) (pPrOzi), have shown to provide high drug loading for a wide variety of drugs. Branched, cyclic, and linear sidechains (*n*-propyl/butyl variants termed nPrOx, nPrOzi, nBuOx, and nBuOzi) have been tested. Of note, recent studies demonstrated a high dependence of the maximum loading capacity (LC) and loading efficiency (LE) of such micelles on both the structure of the polymer and the drug,^46,49,50^ where an exchange of B blocks with structural isomers resulted in drastic changes of the mentioned properties. Superior drug loadings for B blocks with the aforementioned, relatively short B block sidechains underline that simply increasing hydrophobicity of the inner polymer blocks does not necessarily improve drug uptake.^51^ Assessing polymer-drug compatibility through solubility parameters obtained by group contribution methods has repeatedly provided only very limited predictability.^49,50^ Furthermore, for the case of coformulations containing multiple drugs, synergistic and antagonistic effects have been reported.^52^

While dissecting the driving polymer–drug interactions for explaining mechanistically the observed differences in LC and LE remains subject to future studies, a recent chemoinformatics-driven study by Alves *et al.*(35) demonstrated the potential of applying QSPR modeling techniques to predict micelle drug loading. Using the so-called SiRMS descriptors (simplex representation of molecular structure) designed to capture the chemical nature of polymer-drug mixtures by defining smaller fragments of the molecules,^53^ they generated prediction models that were successfully used to improve the experimental hit rate of finding new formulations with high drug loading, as was determined on a subset of eight selected hit compounds from virtual screening.

While the data set used by Alves *et al.*(35) was already quite large (around 400 formulations) and chemically diverse with respect to the tested drugs, it mainly included polymers consisting of the same A and B block monomers (MeOx and mostly nBuOx). Since then, multiple new studies with additional formulation data regarding these DDS have been published, including a variety of drugs, polymer compositions, and information on long-term stability to evaluate shelf life. Thus, in this work, we aim to collect the experimental data of previous publications, combined with in-house data, in order to generate, with a significantly extended data set, an open-access prediction tool that can readily be used by other formulation scientists to evaluate whether these types of polymers could provide an enhanced solubility profile for a potential new drug candidate. Virtual screening of known compounds further provides an openly accessible database of potential use cases with regard to already known, poorly soluble drugs.

## Methods

### Formulation Database

**Figure 2:**
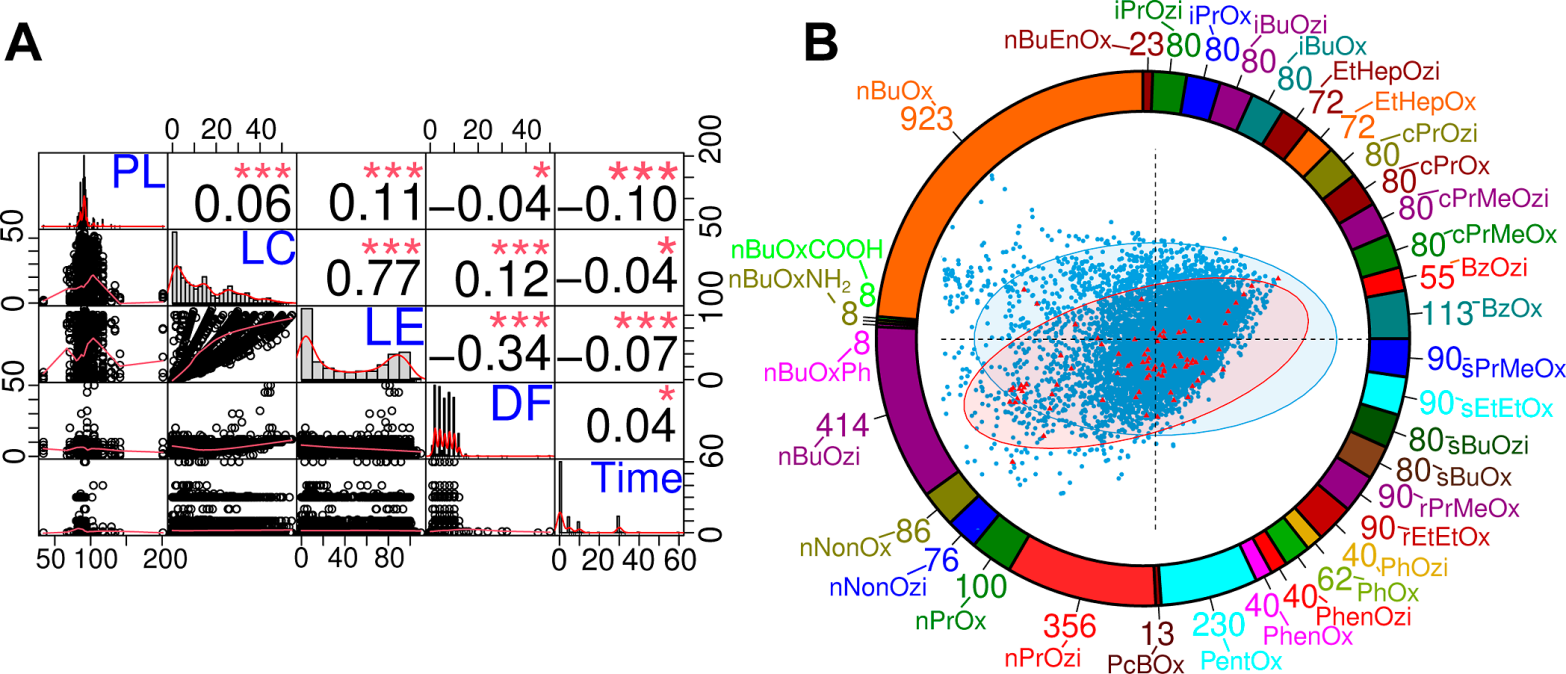
(A) Correlation matrix of several properties illustrating the distributions and correlations of polymer lengths (PL), LC, LE, DF and the time point of solubilization measurement within the formulation database. The matrix includes bivariate scatter plots for each combination of these properties, with fitted lines in red (bottom left), histograms of the distributions of the single properties (diagonal), as well as Pearson correlation coefficients for correlations (top right, with stars signaling *p*-values). (B) Distribution of hydrophobic B block types within the data set (names are defined in Table S1) shown as the outer pie chart. Within the pie chart, a principal component analysis (PCA) with concentration ellipses (confidence level: 95%) of all drugs from DrugBank^69−73^ with moderate to poor solubility (<10 g/L, colored blue) and compounds from the data set (colored red) is illustrated. The PCA was performed on various scaled molecular descriptors in order to compare the properties of the drugs present within our database with the relevant drug space. Descriptors were calculated *via* mordred^74^ (molecular weight, SLogP, number of rotatable bonds, rings, heavy atoms, hydrogen bond donors, and acceptors).

Solubilization data of drug–polymer mixtures from several previous publications^35,46,49−52,54−64^ were combined together with in-house data^65−67^ to create a formulation database of 3700 experimental data points. These are listed in Table S1, which also includes polymer and monomer names used throughout this article. Solubilization data were collected from these publications either by extracting or by calculating the mean values of solubilized drug from given tabulated data or graphically from plots using WebPlotDigitizer 4.6.^68^ The data include a large variation of structures and concentrations of polymers and drugs as well as of the time point for measuring solubilization. The latter allowed for significantly increasing the size of the data set by also including long-term stability information. Furthermore, coformulations of polymers with two drugs were included. Differentiating the chemical structure of monomers and lengths of individual blocks, the data set included 82 different polymer block compositions, with 34 different B block monomer types, 2 A block monomer types (98% pMeOx and 2% pEtOx) and 84 drugs. Figure 2 provides a brief overview of selected properties. As expected, a high correlation between LC and LE values (0.77) is detected, with high values of the former only present for the case of high values of the latter. These properties were calculated based on the amount of solubilized drug (eqs 1 and 2). They describe the relation between the drug feed (DF) (*m*_added drug_, in g/L), the measured solubilization (*m*_solubilized drug_, in g/L), and the polymer feed (*m*_polymer feed_, in g/L), assuming complete solubilization of the polymer. In addition, a negative correlation (−0.34) between the DF and LE values can be observed, as higher LE values are more likely in the case of low drug loadings. Drugs of the data set cover a large proportion of the relevant chemical space in which most drugs with low solubility are located (Figure 2B).
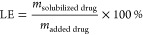
1
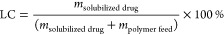
2

### Experimental Settings

**Figure 3:**
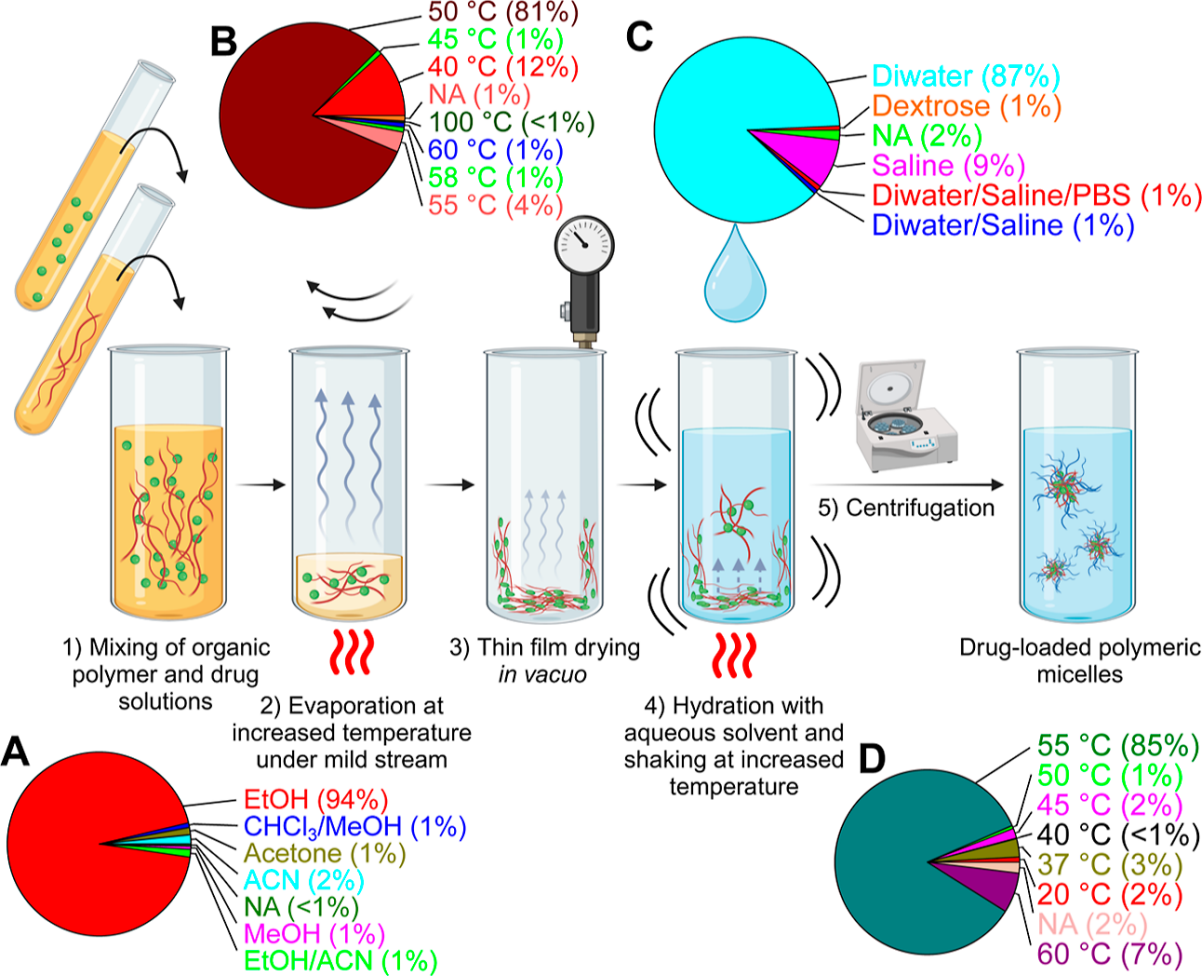
Illustration of the thin-film hydration method used for all formulations (created with BioRender.com). Additional pie charts show the distribution of experimental settings in the whole data set regarding (A) choice of organic solvent, (B) temperature during evaporation, (C) type of aqueous solvent used for dissolution, and (D) temperature during hydration. The majority of formulations was done using ethanol (EtOH), with an increased temperature of around 50–60 °C during evaporation, and distilled water (Diwater) for hydration at the same elevated temperature range. NA refers to instances with missing information. For more details on individual experiments, see Table S1.

While data were obtained from multiple studies, all referenced works followed the thin-film hydration method for drug formulation (Figure 3).^55^ A general protocol in line with most experiments can be described as follows: separate stock solutions of polymer and drug dissolved in volatile organic solvents (usually ethanol with 2–20 g/L solute) were mixed in the desired ratio, and subsequently, the solvent was evaporated under a mild stream of nitrogen, argon, or air at increased temperature (mostly 50–60 °C). This created a thin layer of a polymer–drug blend, from which remaining traces of solvent were removed *in vacuo* (≤0.2 mbar). The resulting dry film was then dissolved with a (37 °C preheated) aqueous solvent (distilled water or buffered saline). This dissolution process was facilitated by shaking the batches at around 1250 rpm for several minutes at a similarly high temperature, as was used during evaporation. The nondissolved drug was subsequently removed from the resulting solution *via* centrifugation at around 10,000 rpm for up to 5 min or filtration. Quantification of the amount of solubilized drug was performed *via* subsequent high performance liquid chromatography (HPLC) or UV–vis absorption experiments.

Analogously as done in the study of Alves *et al.*,^35^ information on the specific experimental settings (summarized in Figure 3) of each individual formulation experiment regarding the chosen organic solvent, the volume of the mixture before evaporation, the type of aqueous hydration solvent, the chosen temperature during evaporation and hydration, and the time point for solubilization measurement is listed in Table S1.

Besides data from several publications, we also collected in-house data from past experiments that were obtained analogously *via* thin-film hydration. This includes 90 data points for solvation of triamcinolone acetonide using polymers with BzOzi, nPrOzi, nBuOzi, BzOx, PentOx, and nBuOx B blocks,^66^ 240 data points regarding solvation of cannabidiol and celecoxib with polymers containing various aromatic B blocks (PhenOx, BzOx, PhOx, PhenOzi, BzOzi, and PhOzi),^65^ and 120 experiments on the solvation of efavirenz and indomethacin with polymers consisting of nBuOzi B blocks and either MeOx or EtOx A blocks (see Table S1 for details on the exact polymer compositions).^67^

Formulations of triamcinolone acetonide were done by mixing ethanolic solutions of polymers (20 g/L) with drug (5 g/L) and subsequent solvent removal at 50 °C, using a water bath and air flow for 10 min. After storage *in vacuo* for 20 min, samples were solvated using 150 μL of 37 °C preheated distilled water. The resulting mixtures were shaken for 15 min at 55 °C and centrifuged for 5 min at 9000 rpm. Experiments for loading of cannabidiol and celecoxib were performed by using 10 g/L ethanolic polymer and drug solutions. Mixtures with desired ratios were evaporated within a water bath at 50 °C under air flow conditions. The resulting polymer–drug layer was stored *in vacuo* (5 mbar) and subsequently hydrated with 37 °C preheated distilled water. The solution was then incubated at 55 °C and shaken at 1150 rpm for 15 min. Mixtures of ethanolic solutions of polymers (20 g/L, 150 μL) with either efavirenz (20 g/L) or indomethacin (10 g/L) were evaporated by using a stream of nitrogen at 50 °C. 300 μL of distilled water was used for hydration, and the solutions were subsequently shaken for 12 min at 1100 rpm and 37 °C. This was followed by centrifugation at 10,000 rpm for 5 min to remove any nondissolved drug. Quantification of the loaded drug was performed *via* HPLC for triamcinolone acetonide, cannabidiol, celecoxib, and efavirenz and *via* UV–vis for the case of indomethacin.

### Model Descriptors

**Figure 4:**
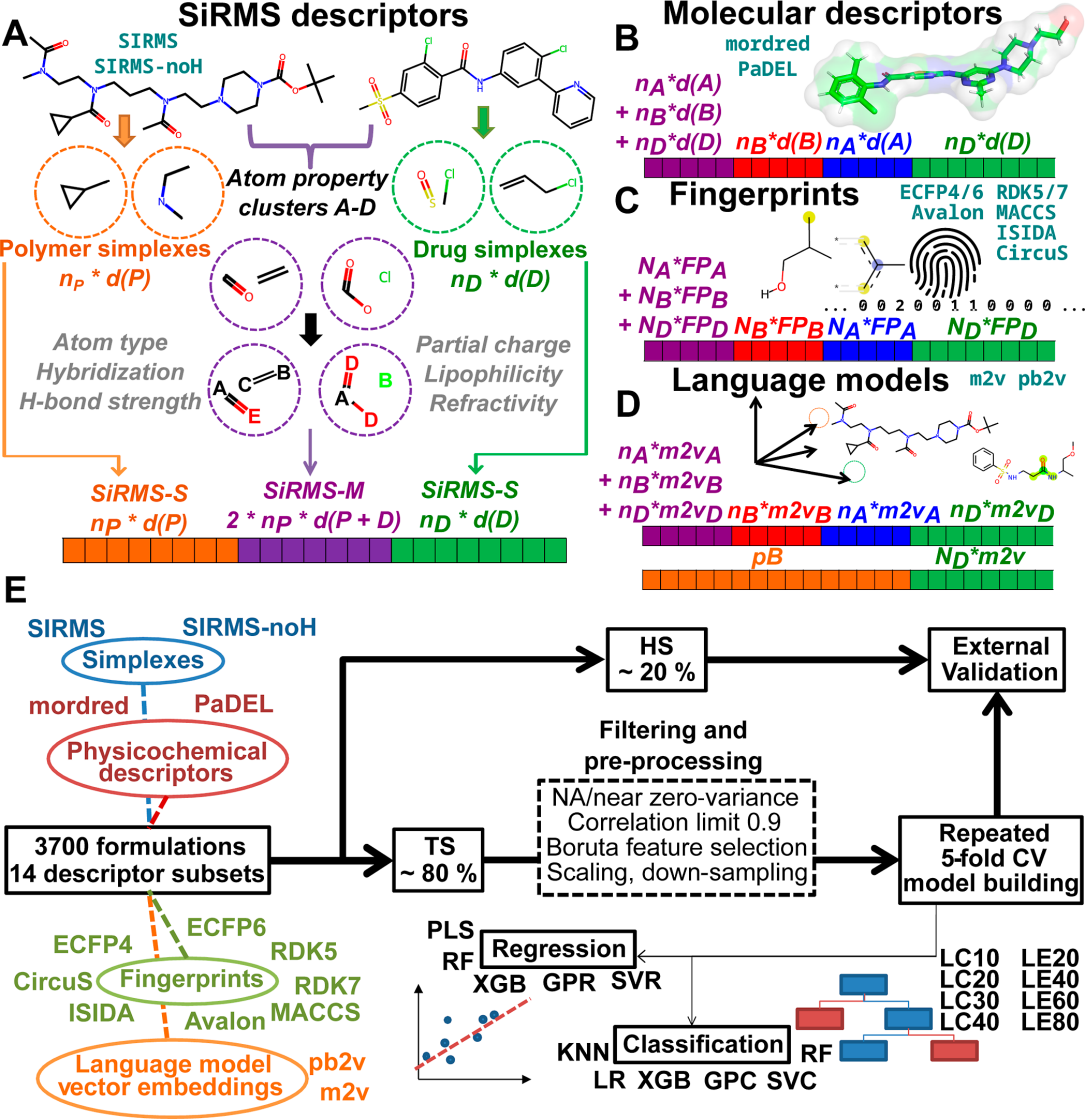
Illustration of computed descriptors, weighted either by molar fractions *n* or number of repeating units and drugs *N* per polymer chain. (A) Determination of polymer, drug (SiRMS-S), and mixture simplexes (SiRMS-M) representing fragment combinations from both molecules. (B) Molecular descriptors are calculated *via* mordred and PaDEL. (C) Extended connectivity, RDKit, Avalon, MACCS, ISIDA, and CircuS fingerprints (FP) encode different substructures and are summed up for mixture-specific descriptors. (D) Polymers and drugs are located within higher dimensional vector spaces constructed by pretrained language models mol2vec (m2v) and polyBERT (pB). Orange, blue, red, and green boxes indicate descriptors for individual components (polymers, A/B blocks and drugs), and violet boxes represent mixture-specific features calculated from all constituents. (E) Model building workflow, involving TS/HS data set splitting, several preprocessing steps, regression, and classification model generation, as well as external validation *via* the HS.

In order to describe polymer–drug mixtures, various molecular features were considered as input variables for model building (Figure 4). Similar to the previous study of Alves *et al.*,^35^ in order to describe mixtures within a QSPR modeling framework, SiRMS descriptors were generated. This technique was developed by Kuz’min *et al.*(75−78) and was successfully used for a variety of QSPR modeling approaches including mixtures^79^ and reactions.^13^ Hereby, all possible tetraatomic subgraphs (called simplexes) describing the chemical nature of smaller unbound and bound fragments of the relevant molecules within a mixture are counted; these numbers represent the simplex descriptor values for subsequent model building (Figure 4A). Mixture simplexes (SiRMS-M) containing fragments from polymers and drugs are weighted by the doubled molar fraction of the minor micellar component, which, for most mixtures, represents the polymer. All simplexes composed of fragments of single molecules (SiRMS-S) are weighted by their respective molar fraction. To account for coformulations, simplexes involving up to three unbound fragments were taken into account. Fragments were discriminated based on whether they belong to the (1) polymer, the (2) first drug for which loading properties were measured, or, within coformulations, the (3) second drug.

For generation of simplexes, different properties were taken into account during clustering of atoms into different subgroups A–D. The HiT QSAR software previously used by Alves *et al.*(35) is based on a graphical interface with slow calculation speed and requires a ChemAxon license to calculate certain atomic properties. In order to develop an open-source tool designed for large-scale screening purposes, we utilized the SiRMS.py tool that was also used in a recent study of Rakhimbekova *et al.*(36) for these polymers. Lipophilicity (*logp*: A ≤ −0.5 < B ≤ 0 < C ≤ 0.5 < D) and refraction (*mr*: A ≤ 1.5 < B ≤ 3 < C ≤ 8 < D) were calculated based on Crippen’s approach^80^ implemented in RDKit 2022.9.5.^81^ Clustering for these properties was performed with the same thresholds used by Alves *et al.*(35) In analogy to this study, SMILES codes of drugs and small pseudotrimers, comprising each monomeric building block and termini once, were used for the generation of SiRMS descriptors. As only tetraatomic simplexes are counted within this approach, the usage of pseudotrimers, with the molar ratios of the actual full-length polymer-drug complexes, captures all relevant fragments but also assumes similar monomer ratios (A/B monomers ≈ 2:1). Atom number labelings were included in combination with hybridization states (*e.g.*, the labeling 6 > sp^2^ in our SiRMS descriptor set represents a sp^2^-hybridized carbon atom), determined *via* the recently published software tool Jazzy 0.0.11,^82^ which itself is built on kallisto 1.0.9.^83^ This tool was further used to compute an extended set of atomic properties: electronegativity equilibration charges (*eeq*: A ≤ −0.28 < B ≤ 0 < C ≤ 0.28 < D, corresponding to cutoffs used by ISIDA^84^), atomic-charge dependent dynamic atomic polarizabilities (*alp*: A ≤ 6 < B ≤ 9 < C ≤ 12 < D), as well as charge-dependent hydrogen bond acceptor and donor strengths (*sa*, *sdx* and *sdc*: A ≤ 0.5 < B ≤ 0.75 < C ≤ 1 < D), where a value of 1 corresponds to the strength of a water H-bond. The SiRMS descriptor set was calculated with and without (SiRMS-noH) consideration of explicit hydrogen atoms.

**Figure 5:**
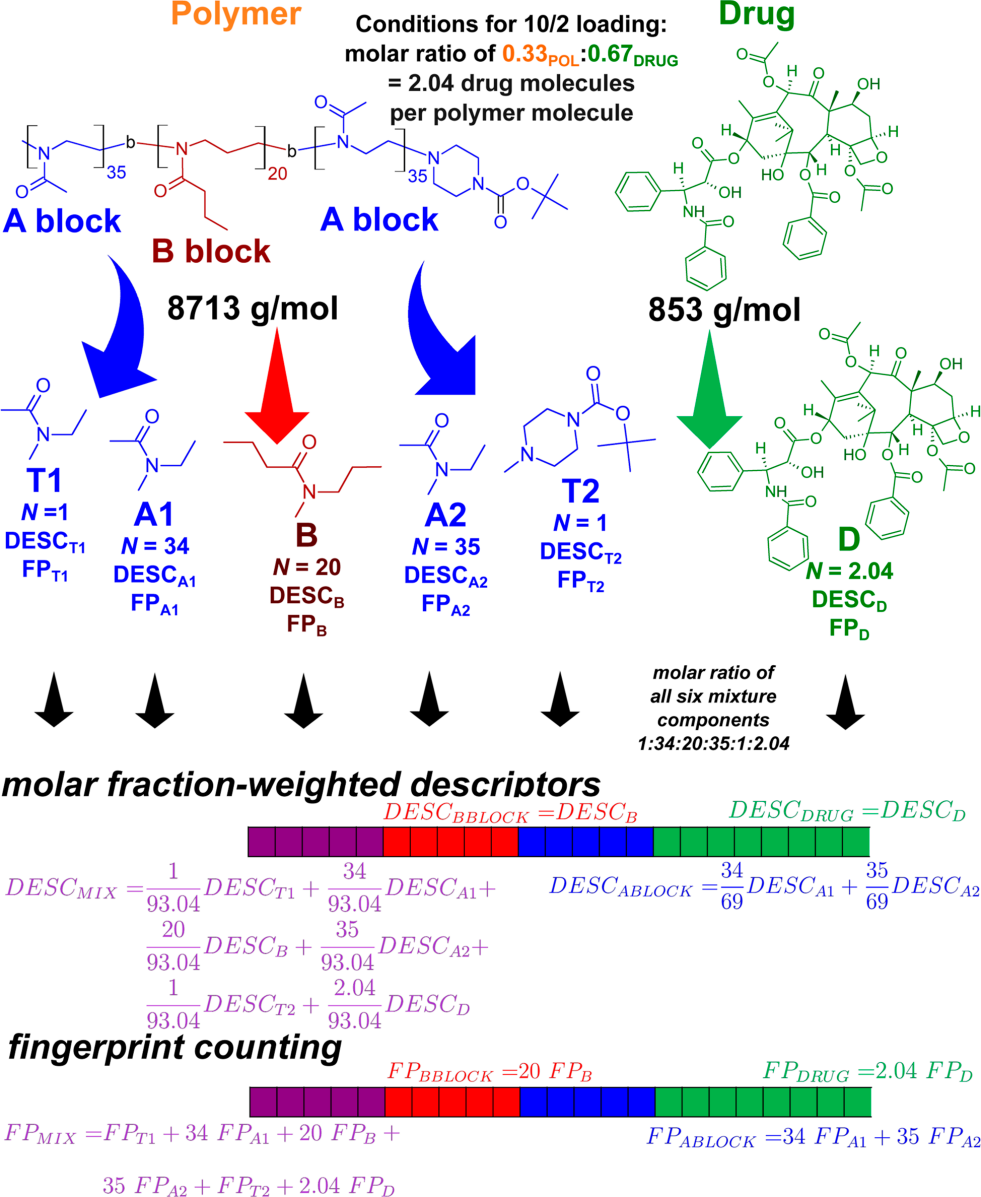
Example of a calculation process for mixture-specific features (Figure 4B–D), shown for the case of paclitaxel loaded into A-nPrOzi-A micelles with a 10/2 polymer-drug mass ratio. Descriptors (*DESC*) and fingerprints (*FP*) were first computed individually for all drug molecules (*D*) and capped building blocks (*T*1, *A*1, *B*, *A*2, and *T*2). Using the mass ratio and block lengths, molar-weighted descriptors (*DESC*_MIX_) as described in eq 3 and count fingerprints (*FP*_MIX_) were computed. SiRMS descriptors (Figure 4A) use pseudotrimers as input instead, as described in the text.^35^

In addition to the SiRMS descriptors, we calculated classical molecular descriptors usually used for QSPR studies of small molecules. For this purpose, SMILES codes of the relevant drugs and monomeric building blocks (example given in Figure 5), capped with a hydrogen on one and a methyl group on the other end (similarly as performed in previous polymer QSPR studies^15,16^), were first loaded into RDKit and 3D structures were optimized *via* the MMFF94s force field.^85^ The monomer nitrogens were capped with methyl groups instead of hydrogens to more closely resemble the tertiary substitution scheme present within the polymer backbone. Subsequently, all 2D/3D molecular descriptors (*DESC*) implemented in mordred^74^ and PaDEL^86^ were calculated (Figure 4B). In order to transform these to features describing mixtures, a similar approach as previous QSPR modeling works was applied:^11,16^ they were weighted according to their molar ratios *n* within the mixture of interest, calculable as  with *N* number of drugs or monomer units of type *i* and *N*_total_ drugs and repeating units in the whole mixture per polymer chain. *N*_total_ in turn can be derived from the amount of A and B monomer units in addition to two terminal groups and the amount of drug per polymer, calculable by the given polymer–drug mass ratio and the respective molecular weights *MW*_polymer_ and *MW*_drug_. Different (nonlinear) combinatorial approaches have been proposed for using such descriptors for mixtures;^11^ in this work, we used a simple linear combination. Thus, individual descriptors for the A and B block repeating units (*DESC*_*A*1/*A*2_/*DESC*_*B*1/*B*2_), the beginning and terminal groups (*DESC*_*T*1/*T*2_), as well as the drug molecules (*DESC*_*D*1/*D*2_) were retrieved and subsequently combined into molar fraction-weighted mixture descriptors (*DESC*_MIX_) that summed up these values (eq 3). Separate mixture descriptors of blocks (*DESC*_ABLOCK/BBLOCK_) and drugs (*DESC*_DRUG_) were calculated analogously.
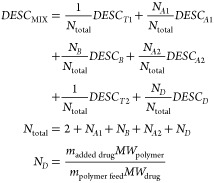
3

Drugs and monomers were used to compute several additional properties through RDKit, representing various methods of describing substructures or the local neighborhood around individual atoms (Figure 4C): extended connectivity fingerprints with radii of 2 and 3 atoms (ECFP4 and ECFP6^87,88^), RDKit fingerprints^81^ with maximum path lengths of 5 and 7 (RDK5 and RDK7), Avalon fingerprints,^89^ and MACCS keys.^90^ Furthermore, ISIDA fragments (maximum length of linear and atom-centered fragments = 5)^84^ and CircuS descriptors (up to a radius of 4)^91^ were obtained through CIMTools and CGRTools,^92^ as implemented within ChemInfoTools.^91^ Because increasing the fingerprint size to reduce the amount of potential bit collisions has shown to improve model performance,^93^ the standard fingerprint size was increased to 16,384 for ECFP and RDK fingerprints. As all of the calculated fingerprints resemble count vectors, in order to compute mixture-specific fingerprints, the counts on each bit of the relevant structures were multiplied by their respective number (number of A/B monomers in one polymer molecule and number of drug molecules per polymer chain, Figure 5 bottom) and subsequently summed up. At last, SMILES codes were also used to generate molar fraction-weighted mol2vec (m2v) vector representations, based on circular Morgan fingerprints with radii up to 1.^94^ Hereby, the molecules are located in a dense, 300-dimensional space in which similar substructures of the chemical space are located in close proximity (Figure 4D). These vector embeddings are based on an unsupervised machine learning procedure on the ZINC database (19.9 million compounds^95^) *via* the natural language processing algorithm word2vec.^96^ Mol2vec vectors were previously concatenated with ProtVec vectors^97^ to construct so-called PCM2vec fingerprints, conveying information on a small molecule compound and its protein target simultaneously and thereby improving predictive performance.^94^ Thus, besides using m2v fingerprints alone, we followed a similar concept by concatenating m2v vectors of drugs with 600-dimensional, polymer-specific polyBERT fingerprints that are based on a language model pretrained on 100 million PSMILES strings and were previously used for polymer property predictions.^24^ These concatenated vectors were termed pb2vec (pb2v).

In addition to the computed mixture descriptors, the following properties were added to all descriptor sets: drug and polymer feeds (in g/L), the time point of solubilization measurement (in days), the number of monomers within each block, molar fractions of each micellar component, and numbers of drugs per polymer chain. While initially we included additional experimental conditions (hydration temperatures and solvent types, relevant to the models of Alves *et al.*(35)), as the database was continuously extended, variations in these properties reduced drastically, as a large majority of formulations from all other publications (compare Figure 3) was reported with the same experimental settings (ethanol evaporation at ∼ 50 °C and hydration with distilled water at ∼ 55 °C) and, furthermore, was lacking information regarding the chosen solvent volume before evaporation, utilized in Alves *et al.*(35) (Table S1). In accordance with this large homogeneity of experimental settings found within our extended data set, preliminary test runs showed these properties to be filtered out *a priori* during the preprocessing and feature selection steps.

### Data Preparation and Model Building

The initial data set of 3700 formulations was used for generating regression models for LC and LE values, as well as classification models for four different threshold values, respectively (LC ≥ 10, 20, 30, or 40%, as well as LE ≥ 20, 40, 60, or 80%), using the R package caret 6.0–91^98^ (Figure 4E). For this purpose, formulations were first split by stratified random sampling into a training (TS, 80%) and an external holdout set (HS, 20%), retaining a similar class ratio within the HS as within the whole data set. For each splitting process during this work, we followed the ‘’mixtures-out” approach,^99^ ensuring that all mixtures encompassing the same types of A blocks, B blocks, and (coformulated) drugs, differing only in polymer-drug mass ratios, block lengths or measurement times, were kept within the same set to prevent data leakage. Within the TS, features of high correlation (correlation limit: 0.9) as well as those containing (near zero) variance or missing values were first filtered out. Next, the Boruta algorithm was used to perform initial feature selection on the TS. Hereby, mean decrease accuracy values of real features and shuffled variants thereof are assessed for initial random forest (RF) models in order to iteratively remove descriptors with low Z scores.^100^ The reduced descriptor subset was then used to generate various commonly used regression and classification model types implemented in caret.

For regression tasks, we generated partial least-squares (PLS), RF, XGBoost (XGB), and multivariate linear regression models as well as support vector (SVR) and Gaussian process (GPR) regressions using a radial basis function as kernel. For classification tasks, in addition to RF, XGB, support vector classification (SVC), and Gaussian process classification (GPC) models, we tested the K-nearest neighbor and logistic regression (LR) algorithms. For all classification models, 5-fold cross-validation (CV) with 20 repeats was performed, with feature scaling and centering, as well as the Yeo-Johnson transformation^101^ applied as preprocessing steps. For hyperparameter tuning, the caret grid search with a tune length of 20 was applied (except for the case of XGB models, where the length was reduced to 5). For all regression models, both the number of CV repeats and the tune lengths were reduced to 10; for XGB models, the length was set to 3. The area under the receiver operating characteristic curve (AUC) was used as an optimization metric for classification models and the root-mean-square error (RMSE) for regression tasks. Due to a large class imbalance at higher thresholds, downsampling was performed for classification tasks, which improved model performance during preliminary test runs.

### Model Evaluation

All models were assessed based on CV results and external predictions with regard to the HS. For regression models, RMSE and mean average error values, as well as the coefficient of determination (*R*^2^), were determined. For classification tasks, besides AUC values, additional parameters depending on the amount of true positives (TP), true negatives (TN), false positives (FP), and false negatives (FN) were investigated: sensitivity/recall (Sens, eq 4), specificity (Spec, eq 5), positive predictive value/precision (PPV, eq 6), negative predictive value (NPV, eq 6), balanced accuracy (Acc, eq 8), and the F1 score (eq 9). Furthermore, the overall best models were determined based on the normalized Matthew’s correlation coefficient (nMCC, eq 10). Unlike Acc and F1 metrics, this statistic includes all four elements of a confusion matrix and is considered more reliable for binary classification tasks.^102−104^
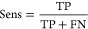
4
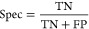
5
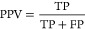
6
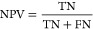
7
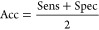
8
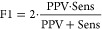
9
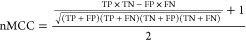
10

Similar to the approach of Alves *et al.*,^35^ for virtual screening of the DrugBank and building of a web tool, formulations were classified as being within the applicability domain (AD) if their Euclidean distance to TS compounds within the multidimensional, scaled descriptor space was below a predefined threshold *D*_cutoff_. The latter was determined by the average distance < *D* > and the corresponding standard deviation *s* based on all mixtures of the TS to its *k* nearest neighbors.^105−107^ Modeling steps for the final models were repeated with Y-randomization in order to assess possible chance correlations (Figure S1).^108^ Feature importance was assessed by computing Shapley (SHAP) values *via* the KernelSHAP algorithm.^109,110^ Stemming from game theory,^111^ these values evaluate the contribution of descriptors to single model outcomes taking into account interactions between the different features.

### Virtual Screening

The best models were used for a screening process with regard to known drug molecules. Compounds present within DrugBank 5.1.10 (https://go.drugbank.com/)^69−73^ were first selected based on the ALOGPS-derived solubility^112^ being below 10 g/L. This includes all (very) slightly soluble to practically insoluble drugs (as defined by the USP^113^). For the whole set of the remaining 9462 molecules, mixture descriptors were calculated, given hypothetical formulations with 5 different polymers present within the formulation database of this study, covering the most common B blocks (A-nPrOx-A,^49^ A-nPrOzi-A, A-nBuOx-A and A-nBuOzi-A^50^), as well as one aromatic B block type (A-BzOx-A^64^). For all of the 47,310 theoretical formulations, polymer feeds of 10 g/L and DF of 6 g/L with immediate solubilization measurement (0 days) were selected as the experimental conditions. Predictions from all eight final classification models (LC10-40, LE20-80) were calculated and further assessed based on evaluation of the AD.

## Results and Discussion

### Model Performance

Regression and classification models for predicting LC and LE values of drug-loaded pOx/pOzi polymer micelles were built based on various mixture-specific molecular descriptors. Classification included investigation of eight different thresholds (LC10, LC20, LC30, LC40, LE20, LE40, LE60, and LE80). Results for all tested model and subset combinations are listed in Tables S2 (regression tasks) and S3 (classification tasks). The naming scheme follows labeling of the chosen subset, the threshold, and the model type (*e.g.*, rdk7-LC10-RF = RDK7 fingerprints-based RF model for a threshold LC value of 10%). With regard to regression, the best models (based on RMSE values) were generated using RDK7 fingerprints and achieved *R*^2^_CV_ values of 0.46 (LC-SVR model, Figure S2A) and 0.56 (LE-RF model, Figure S2B). These statistics do not meet the recommended requirements for QSPR regression modelability^114^ and suggest that a classification approach with multiple thresholds, as performed by Alves *et al.*,^35^ should be preferred for this data set. Thus, the following discussion focuses on the performance of the investigated classification tasks.

**Figure 6:**
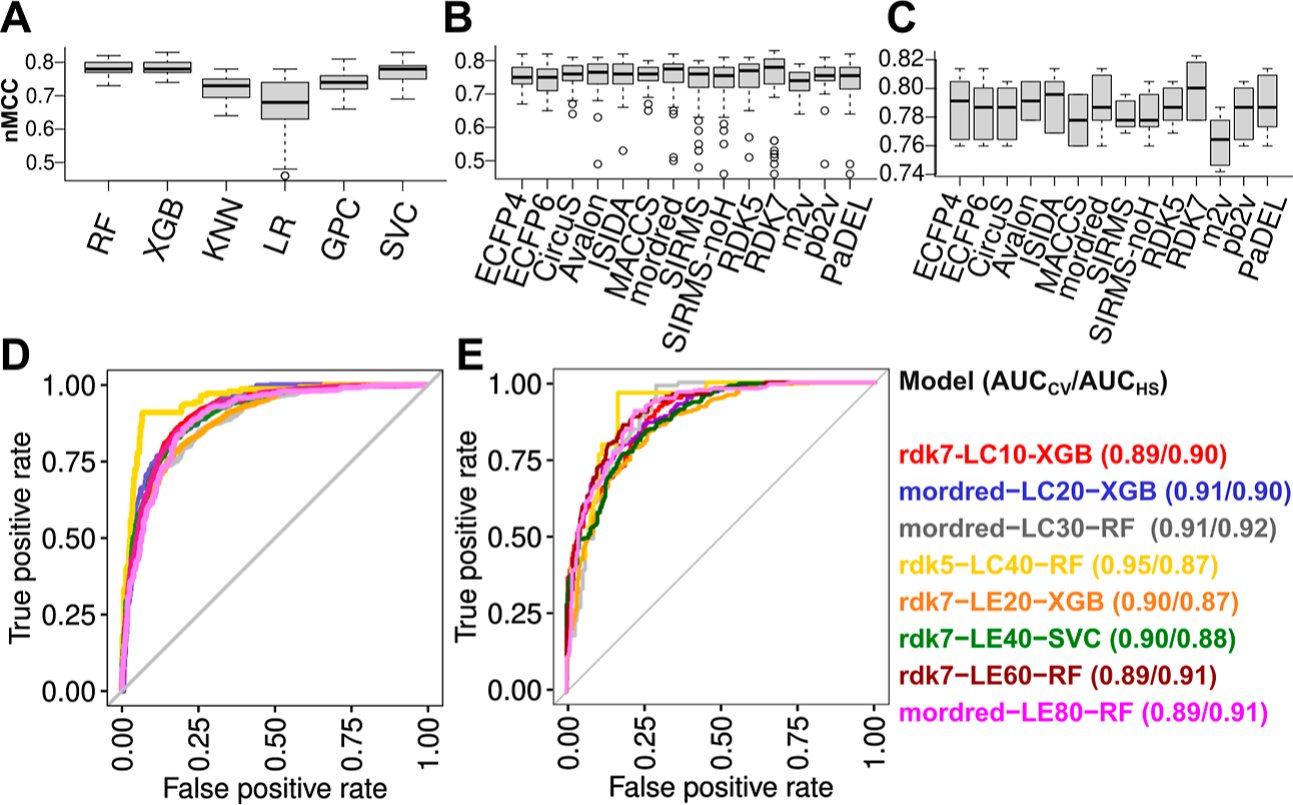
Boxplots of median nMCC_CV_ scores depending on (A) model type, (B) chosen descriptor subset, or (C) chosen descriptor subset with only selecting the best model for each threshold. (D) AUC_CV_ and (E) AUC_HS_ curves for the final models listed in Table 1.

Overall, tree-based model types (RF and XGB) and support vector classifiers achieved the best nMCC_CV_ scores, reaching median values of 0.78 (Figure 6A). When the different descriptor subsets were compared, RDK7 fingerprints, similar to the results of the regression models, performed best, with median nMCC_CV_ values of 0.78 across all model types (Figure 6B) and 0.81 for the best models of each investigated threshold (Figure 6C). However, differences between median values of most subsets are small, suggesting that each set is able to capture, to some extent, important structural elements for determining drug loading. The descriptor subset m2v performed worst, which might be at least partly due to the way the vector embeddings were mixed (weighted by molar fractions). Substituting m2v vectors with pb2v vector embeddings for the polymer of each formulation increased performance. We hypothesize that these language model-based approaches could be further improved, *e.g.,* by adjusting the Morgan circular fingerprint radius for m2v descriptors or accounting for the invariance to block copolymeric compositions of the polyBERT fingerprints (as discussed in ref (24)). The calculated SiRMS descriptors did not outperform the more conventional molecular descriptors and fingerprints often used in QSPR studies of small molecules. However, as described in the Methods section, this could partly result from the alternative computation of atomic properties with the aim of developing an open-source prediction tool.

Selecting the best models for each threshold individually provides the CV statistics listed in Table 1 (top half). With a combination of physicochemical descriptors from mordred and RDK fingerprints encoding substructural elements of drugs and polymers, nMCC_CV_ and Acc_CV_ values of around 0.8 and AUC_CV_ values of around 0.9 are retrieved. These fulfill the requirements for classification modelability (Acc > 0.7^115^) and suggest, as opposed to the regression models shortly described above, overall very good predictive performance. Comparing the results to the models of Alves *et al.*,^35^ previously trained on a subset of nearly 400 formulations, shows an overall similar performance; Acc, Sens, Spec, and NPV values indicate good performance and are all ranging mostly between 0.7 and 0.9. In contrast, our models at higher thresholds, specifically LC30 and LC40, show smaller PPV values, which also lead to lower F1 scores accordingly. This is an observation not detected by high AUC values (Figure 6D), which are often used as a single evaluation metric. However, as our null models indicate (representing trivial classification models used as baseline estimations, assigning all instances to the positive class), a large data imbalance at these thresholds results in a very low prevalence of formulations exceeding these thresholds. In our extended formulation database, at a threshold of LC40, the amount of positive data points equals only about 5% (see the PPV value of LC40-NULL, Table 1). Given a PPV_CV_ statistic of 0.38, a selection based on the rdk5-LC40-RF model increases the chance of TP hits up to 8-fold. Simultaneously, high NPV values suggest that, despite these low PPV statistics, the models efficiently filter out large amounts of truly negative hits and could thus be used as a prescreening tool in the early stages of drug formulation development, reducing the amount of compounds needed to be tested experimentally.

**Table 1:** Statistics of the Best Models for Fivefold CV and Performance on the External HS^a^

CV statistics								
model	nMCC	AUC	ACC	SENS	SPEC	F1	PPV	NPV
**rdk7-LC10-XGB**	**0.80**	**0.89**	**0.80**	**0.81**	**0.79**	**0.81**	**0.80**	**0.80**
Alves-LC10-RF			0.83	0.89	0.77		0.85	0.83
LC10-NULL		0.50	0.50	1.00	0.00	0.68	0.51	
**mordred-LC20-XGB**	**0.82**	**0.91**	**0.83**	**0.81**	**0.86**	**0.75**	**0.71**	**0.92**
Alves-LC20-RF			0.82	0.75	0.88		0.81	0.84
LC20-NULL		0.50	0.50	1.00	0.00	0.46	0.29	
**mordred-LC30-RF**	**0.78**	**0.91**	**0.83**	**0.77**	**0.88**	**0.62**	**0.52**	**0.96**
Alves-LC30-RF			0.85	0.82	0.89		0.77	0.92
LC30-NULL		0.50	0.50	1.00	0.00	0.25	0.14	
**rdk5-LC40-RF**	**0.78**	**0.95**	**0.92**	**0.91**	**0.93**	**0.54**	**0.38**	**1.00**
Alves-LC40-RF			0.83	0.70	0.96		0.83	0.93
LC40-NULL		0.50	0.50	1.00	0.00	0.09	0.05	
**rdk7-LE20-XGB**	**0.83**	**0.90**	**0.82**	**0.84**	**0.81**	**0.84**	**0.85**	**0.80**
LE20-NULL		0.50	0.50	1.00	0.00	0.71	0.56	
**rdk7-LE40-SVC**	**0.83**	**0.90**	**0.83**	**0.82**	**0.84**	**0.82**	**0.81**	**0.84**
LE40-NULL		0.50	0.50	1.00	0.00	0.63	0.46	
**rdk7-LE60-RF**	**0.82**	**0.89**	**0.83**	**0.84**	**0.81**	**0.78**	**0.74**	**0.89**
LE60-NULL		0.50	0.50	1.00	0.00	0.55	0.38	
**mordred-LE80-RF**	**0.78**	**0.89**	**0.79**	**0.74**	**0.85**	**0.69**	**0.64**	**0.90**
Alves-LE80-RF			0.76	0.76	0.76		0.75	0.76
LE80-NULL		0.50	0.50	1.00	0.00	0.42	0.27	

HS statistics								
model	nMCC	AUC	ACC	SENS	SPEC	F1	PPV	NPV
**rdk7-LC10-XGB**	**0.80**	**0.90**	**0.80**	**0.81**	**0.79**	**0.82**	**0.83**	**0.77**
LC10-NULL		0.50	0.50	1.00	0.00	0.71	0.55	
**mordred-LC20-XGB**	**0.80**	**0.90**	**0.80**	**0.73**	**0.87**	**0.72**	**0.72**	**0.88**
LC20-NULL		0.50	0.50	1.00	0.00	0.47	0.31	
**mordred-LC30-RF**	**0.75**	**0.90**	**0.81**	**0.81**	**0.82**	**0.57**	**0.44**	**0.96**
LC30-NULL		0.50	0.50	1.00	0.00	0.26	0.15	
**rdk5-LC40-RF**	**0.71**	**0.92**	**0.84**	**0.80**	**0.88**	**0.38**	**0.25**	**0.99**
LC40-NULL		0.50	0.50	1.00	0.00	0.09	0.05	
**rdk7-LE20-XGB**	**0.78**	**0.87**	**0.78**	**0.75**	**0.80**	**0.79**	**0.84**	**0.70**
LE20-NULL		0.50	0.50	1.00	0.00	0.73	0.58	
**rdk7-LE40-SVC**	**0.79**	**0.88**	**0.79**	**0.81**	**0.77**	**0.78**	**0.75**	**0.83**
LE40-NULL		0.50	0.50	1.00	0.00	0.63	0.46	
**rdk7-LE60-RF**	**0.83**	**0.91**	**0.83**	**0.80**	**0.86**	**0.80**	**0.79**	**0.86**
LE60-NULL		0.50	0.50	1.00	0.00	0.58	0.40	
**mordred-LE80-RF**	**0.79**	**0.91**	**0.80**	**0.74**	**0.86**	**0.72**	**0.69**	**0.89**
LE80-NULL		0.50	0.50	1.00	0.00	0.46	0.30	

a Additionally, available statistics for previously published models of Alves *et al.* generated for a subset of around 400 formulations,^35^ as well as null models as reference are listed. The latter assume positive classifications for all instances, thus providing baseline probabilities for comparison.

In addition to CV during model building with the TS (3050 formulations), the performance of the final models was assessed *via* the external HS (650 formulations) filtered out at the beginning of the modeling workflow (Table 1, bottom half). Statistics and AUC curves (Figure 6D) are very similar to the CV results, further validating that the models can provide a similar predictive performance when new data are obtained. High Acc_HS_ values around 0.8 are retained, which indicates that the models reach a similar performance to those of Alves *et al.*,^35^ which were tested on eight additionally performed experimental data points instead of a prefiltered HS and reached a correct classification rate of 75%. In addition to the selected final models, predictions for the best subset-model type combination determined above (RF models with the RDK7 subset, termed RDK7-RF) are listed in Table S2 in the beginning. These models reach nearly the same degree of overall balanced accuracy (Acc_HS_ = 0.75–0.83, see Table S3), while requiring calculation of only one descriptor type and avoiding the complexity of gradient-based XGB models.

### Model Interpretation

Modeling results show that by using RDK fingerprints and/or physicochemical descriptors, models can be built which sufficiently discriminate between formulations based on loading properties. By analyzing SHAP values, it is possible to determine the most important features across various model types and interpret the results, as, *e.g.,* previously shown for predictions of formulation printability.^116^ Thus, in the following section, we describe briefly the most relevant descriptors for selected models.

With respect to experimental descriptors, SHAP values of all individual models (Figure S3) show that the time point of solubilization measurement plays an important role, in particular for models with lower thresholds (LC10, LC20, LE20, and LE40). As expected, larger values (corresponding to long-term stability measurements) are associated with negative predictions. This suggests that drug loadings of formulations with lower initial LC and LE values are also affected more by long-term storage than those corresponding to ultrahigh loading properties. Furthermore, the DF represents another important experimental property for all models. As expected, higher values are associated with more positive predictions for LC and more negative predictions for LE values. This conforms to the correlation shown in Figure 2: high LC values, depending on the weight of all micellar components, are only possible at higher DF, whereas high LE values, depending only on the fraction of solubilized drug, are naturally more common at lower DF. Thus, predictions of the models are largely associated with these dependencies: LC models are more likely to predict positive hits for higher DF, while for LE models, the conditions are vice-versa.

**Figure 7:**
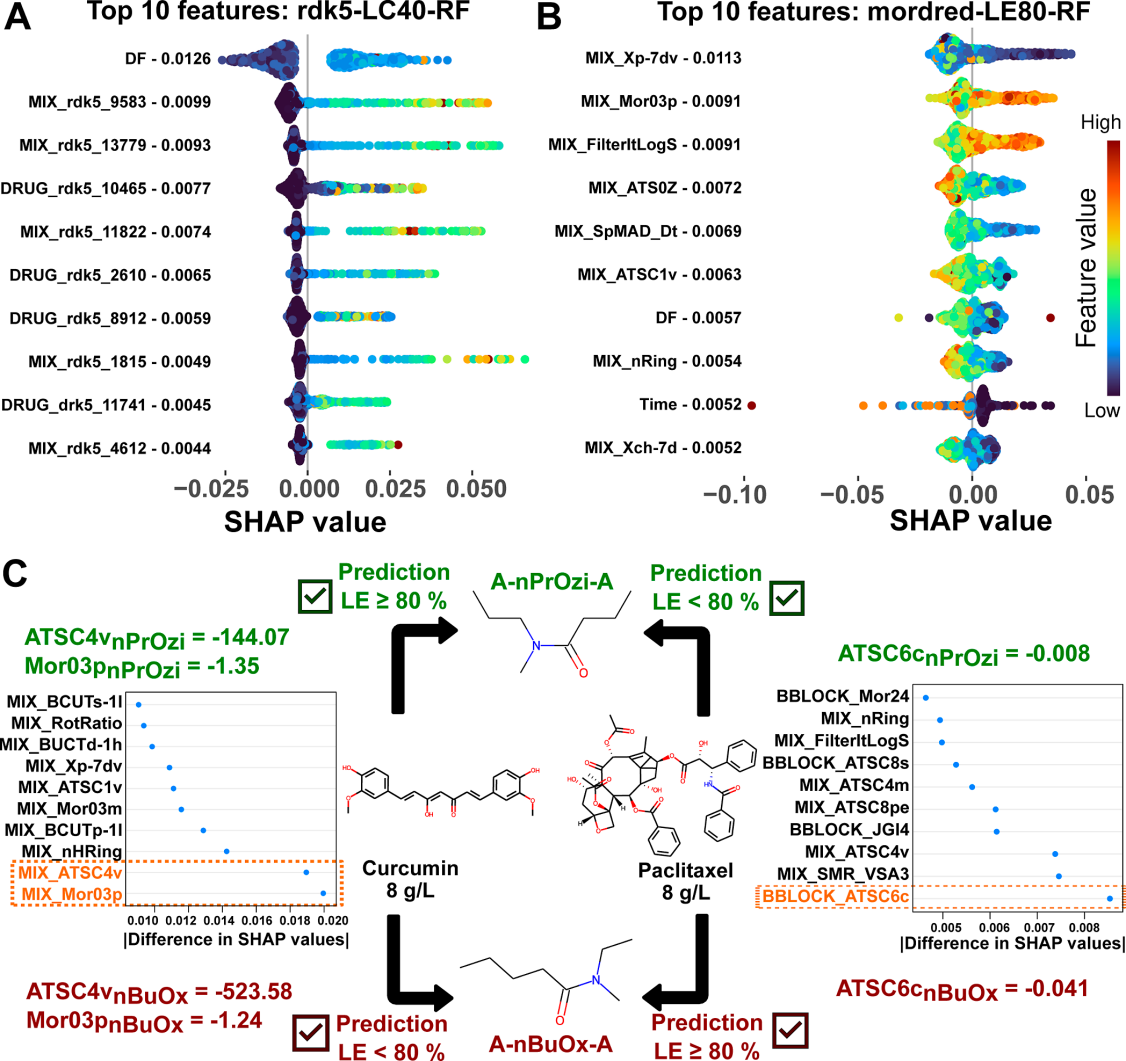
Top 10 features of models (A) rdk5-LC40-RF and (B) mordred-LE80-RF from Table 1, sorted according to mean absolute SHAP values (listed next to each descriptor name). Each data point represents a formulation of the TS and is colored according to the respective descriptor value. Negative SHAP values correspond to formulations where the corresponding feature supports a negative prediction (threshold not passed), whereas points where the descriptor value contributes to a positive model prediction (threshold passed) are assigned larger SHAP values. This allows for detection of trends in feature values contributing to high drug loadings. In (C), the top 10 descriptors with the largest absolute differences in SHAP values, where features lead to different outcomes for A-nPrOzi-A and A-nBuOx-A loaded with either curcumin or paclitaxel, are sorted in a dotplot on each side for the respective drugs. Values of the different B blocks are listed for the most important features, marked in orange.

**Figure 8:**
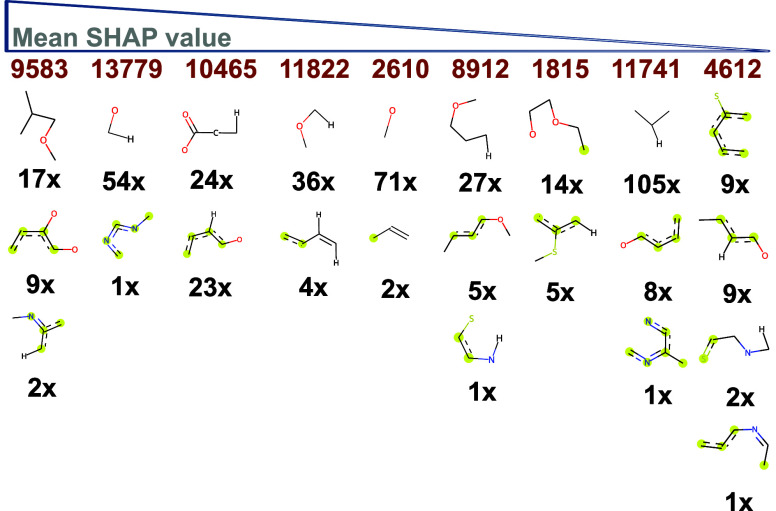
Top 10 RDK5 fingerprints for model rdk5-LC40-RF based on SHAP values. For each bit position, with its number depicted on top in orange, the structural elements responsible for increasing the counts are listed below. Aromatic elements are highlighted in yellow. Numbers beneath each fragment count the unique amount of drugs and monomeric repeating units within the data set that contain the corresponding substructure.

Looking beyond the experimental settings, SHAP values for the most important features of models rdk5-LC40-RF (Figure 7A) and mordred-LE80-RF (Figure 7B) provide insight into structural elements and properties of formulations enabling ultrahigh drug loading. From the RDK5 fragments responsible for increasing the count of the relevant fingerprint bits (Figure 8), it is evident that the rdk5-LC40-RF model predicts high LC values for mixtures containing large amounts of nonaromatic oxygen elements such as hydroxyl and ether groups, either situated adjacent to alkyl groups or directly bound to aromatic rings. Bit 9583 encodes for (alkylated) catechol groups present, *e.g.,* in curcumin, etoposide, diosmin, and erlotinib, but also for lactone moieties in simvastatin and podophyllotoxin. Bits 13,779 and 2610 count many different oxygen moieties, including a large quantity of hydroxyl groups as, *e.g.*, found in curcumin, paclitaxel, rutin, and dasatinib. To smaller degrees, fingerprints also encompass aromatic nitrogens (*e.g.*, bit 4612 for olanzapine) and sulfur atoms bound to aromatic rings. The latter includes sulfonyl groups as found in vismodegib and sulfonamides, *e.g.*, present in celecoxib. Only the descriptor *DRUG*_*rdk*5_11,741 includes a mainly hydrophobic propyl fragment limited to drugs. A higher number of all of these RDK5 fragments contributes to higher loading, as indicated by positive SHAP values. This suggests that the hydrophobic cargo should contain several polar elements, likely for interactions with the amide groups of the polymers.

The amount of hydrogen bond forming functional groups, captured with the majority of these RDK fingerprints, has also been investigated as influential parameter in previous studies^56,117^ for a smaller selection of drugs. While Lim *et al.*(117) discussed electronegative and hydrogen bond-accepting groups as polymer interaction strengthening factors for paclitaxel and vismodegib, the presence of tertiary amides within the polymers should favor interactions with donor groups, *e.g.,* hydroxyl groups encoded with RDK5 bits 13,779 and 2610. However, as we previously observed both experimentally and *in silico*,^45,48^ these micelles contain significant amounts of water. In principle, this can affect micelle morphology^118^ but also enable polymer–drug interactions with acceptor groups *via* bridging water molecules, as partly observed in our recent modeling study.^48^ As we also described for a smaller drug library,^50^ we could not detect a simple correlation of the number of hydrogen bond donors, acceptors, or their ratio with LC or LE values for this extended database. While the probability of drugs with no donor or acceptor groups providing high LC values is low (Figure S4), there are still exceptions. Drugs that can be solubilized with LC values above 30% with no donor groups resemble aromatic molecules: schisandrin A,^64^ clotrimazole, mitotane,^50^ and BT-44.^60^ Overall, the relevant fingerprints suggest that hydrophobic guest molecules need to contain a sufficient amount of polar elements that could enhance loading through coordination of polymer amides beyond more unspecific hydrophobic interactions.

Complementary to structural fragments relevant for rdk5-LC40-RF, the top 10 molecular features of mordred-LE80-RF, as determined by SHAP values, encompass a large amount of topological descriptors that essentially convey information about the molecular shape or the distribution of different properties across the compound (see additional details on these descriptors in the Supporting Information). The molecular connectivity Chi indices *Xp* – 7*dv* and *Xch* – 7*d* depend on the amount of molecular fragments of a length of 7 bonds, whereby the constitutive information conferred by quantitation of these subgraphs is further complemented by electronic information *via* weighting of the vertices by either valence (*dv*) or sigma (*d*) electrons.^119^ Subgraphs of this size are not present within monomeric repeating units of smaller size (*e.g.*, MeOx) and thus the polymer, which has a larger impact on the mixture descriptor for the case of lower DF, generally contributes to lower values that result in positive predictions. The rather abstract nature of such features can be interpreted more easily when directly comparing the Chi indices for different drugs. Curcumin enables ultrahigh loading with, *e.g.,* A-nPrOzi-A and shows very low values for these descriptors (*Xp* – 7*dv* = 0.07). In contrast, we detect the highest indices for steroids (*Xp* – 7*dv* of triamcinolone acetonide = 0.94), comprising a more complex and cyclic molecular skeleton and resulting in lower drug loadings. Values for (centered) *ATS*(*C*) Moreau-Broto autocorrelation descriptors are computed based on different properties (*e.g.*, *Z* = atomic number, *v* = van der Waals volume) taking into account all atom pairs with a certain topological distance (*e.g.*, 1 for *ATSC*1*v*).^120^ As *ATS*0*Z* and *ATSC*1*v* describe small distances, they highly correlate with the molecular weight of a compound; thus, low DF lead to lower values for mixture descriptors of this kind. Larger topological distances consider interactions between more distant atom pairs and can detect differences, *e.g.,* between monomers (see below). Furthermore, we previously used the important descriptor *SpMAD*_*Dt* of the detour matrix, describing all atom–atom distances within a molecule,^121^ for predicting drug–lipid interactions, showing distinct values of this feature for symmetric molecules.^122^ Overall, the relevance of all these topological descriptors suggests that the shape of molecules could play a crucial part in the loading process, as we previously also determined for the case of incorporation of drugs into lipid bilayers: the structurally rigid and long molecule Itraconazole, *e.g.,* adopts unfavorable orientations in presence of cholesterol, as the latter prevents more favorable drug arrangements parallel to the membrane surface.^123,124^

The two descriptors within the top 10 features of mordred-LE80-RF, where higher values are associated with positive predictions, are *Mor*03*p* and *FilterItLogS*. The latter represents a group-based contribution method, similar to logP calculation methods, to compute the solubility of a compound. Thus, the corresponding mixture descriptor represents the average solubility of all constituents of the micelle; a mixture with high average LogS value enables high loading beyond LE values of 80%. At last, *Mor*03*p* is a 3D-MoRSE descriptor that encodes for the distribution of molecular polarizability across the surface of the compound (based on the concept of scattering functions^125^), with curcumin and MeOx repeating units providing both relatively high values. In the study of Hwang *et al.*,^56^ the number of rotatable bonds in drug molecules has been mentioned as additional parameter for drug loading, favoring more flexible compounds. While not listed in the top 10 features based on SHAP values, descriptors *nRot* and *RotRatio* are detected as important properties (within the top 20) for most models that are based on the mordred subset.

We note that besides SHAP values, multiple other ways for estimating the contribution of descriptors exist. For example, feature importance of tree-based models is often assessed by determining the mean decrease of accuracy or impurity.^126^ Furthermore, for investigating the influence of individual descriptors, partial dependence plots^127^ and, more recently, the faster method of accumulated local effects (ALE)^128^ were proposed. The latter is especially suited in the presence of correlated features.^129^ To complement our SHAP analyses, we calculated ALE values for the top 10 SHAP features of models rdk5-LC40-RF (Figure S5) and mordred-LE80-RF (Figure S6), in order to further study the impact of these features on model outcomes isolated from the effects of other descriptors. Similar to the SHAP analysis, positive values are associated with a contribution to positive classification outcomes. All plots show the same general up or downward trends that were indicated by the SHAP plots (Figure 7). As might be expected for classification tasks, changes in ALE values for descriptors of model rdk5-LC40-RF are quite sharp, suggesting the LC threshold to be reached quickly once a certain amount of substructural elements is present within the mixture. Furthermore, ALE values for mordred-LE80-RF show subtle trends that are not readily detectable in the corresponding SHAP plot: for some descriptors, like *FilterItLogS* and *SpMAD*_*Dt*, an initial upward trend in regions of lower values can be observed before a continuous decrease at larger values. Hence, for these properties, there exists not a general trend but rather a certain range that maximizes drug loading.

#### Models Predicting Polymer Selectivity

Our data set extends the formulation library of Alves *et al.*(35) containing a diverse set of drugs particularly in terms of measurements involving additional polymer compositions. We previously determined curcumin to show relatively low loading for A-nBuOx-A (3.2 g/L), but very high loading for A-nPrOzi-A (11.9 g/L) containing a structural isomer as a B block. For paclitaxel, B blocks with butyl sidechains are preferred instead.^52^ Investigating the model outcomes at 8 g/L DF, both cases are classified correctly by mordred-LE80-RF, with the curcumin data point located within the TS used for cross-validated model generation and the paclitaxel data point within the external HS used for additional external validation. Large differences in SHAP values between the systems A-nBuOx-A and A-nPrOzi-A with the same block lengths for both drug molecules are determined, in particular, for topological autocorrelation and 3D MoRSE descriptors (Figure 7C). *ATSC*4*v* and *ATSC*6*c* encode for the distribution of the van der Waals volume and charges, *Mor*03*p* for the polarizability. In contrast to Hansen solubility parameters investigated for drug loading predictions in previous works,^49,50^ all these properties are able to differentiate between the structural isomers nBuOx and nPrOzi, leading to variation in mixture-specific features. Other descriptors, *e.g.*, *Xp* – 7*dv*, do not differ between nBuOx and nPrOzi monomers. Minor differences in values of such features are, however, detected and influence model predictions, as variations in structures of the termini affect the molar ratios from which mixture descriptors are computed. We previously compared the curcumin-loaded micelles using a molecular modeling approach and determined differences in hydrogen bonding dynamics.^48^ Such events are difficult to capture using static descriptors from building blocks. Thus, we must consider that while models achieve overall high accuracy and topological descriptors contribute to assessing polymer selectivities, effects of small structural differences could still require more resource-intensive computational approaches.

### Virtual Screening

**Figure 9:**
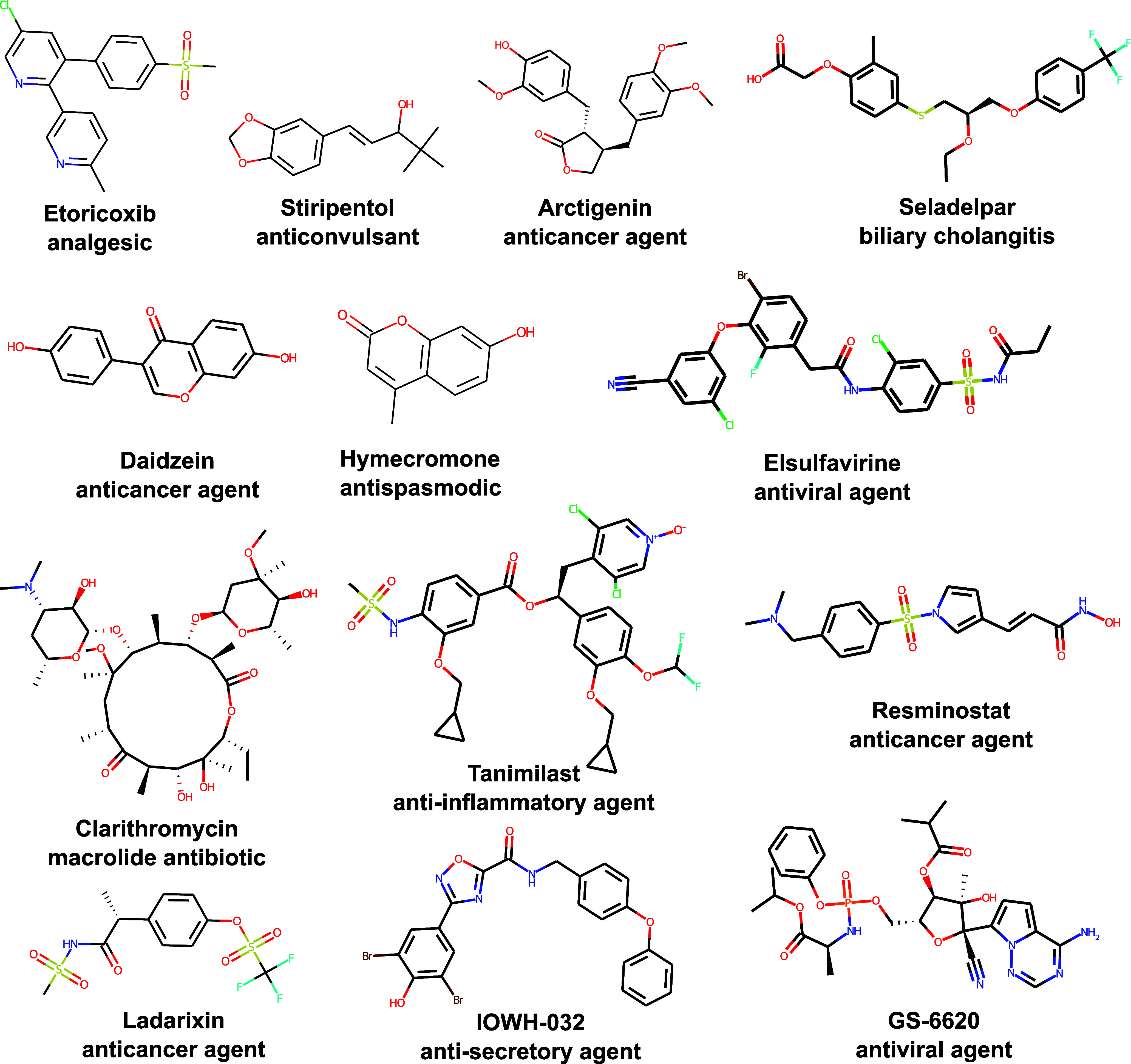
Example compounds for which the final models predicted favorable drug loading properties for pOx/pOzi-based micelles, with at least five thresholds passed at 6 g/L DF.

We used the final models to screen the DrugBank for potential use cases of the most commonly used pOx/pOzi polymers. All results can be found in Tables S4 and S5 (sortable by the amount of model thresholds passed), while Figure 9 shows some examples of compounds predicted to result in favorable drug loading properties, passing at least five of the eight thresholds at 6 g/L DF (including LC ≥ 30 or LE ≥ 60). Besides many taxanes that are structurally closely related to paclitaxel making up a relatively large amount of data points of our formulation database, a variety of compounds across different therapeutic fields are predicted to result in high loading, including, *e.g.,* macrolide antibiotics, analgesics, and anticancer agents. Structures reminiscent of curcumin are also found, containing di- or trimethoxyphenyl moieties or hydroxyphenyl groups, like many (iso-)flavons. The hits also include multiple compounds in clinical trials that have not been formulated with such polymeric systems yet. This includes, for example, ladarixin (CXCR1/2 inhibitor) and resminostat (HDAC-6 inhibitor). Both classes of drugs are investigated for synergistic effects with other anticancer agents, including taxanes,^130,131^ and are considered, *e.g.,* for combination therapies against pancreatic cancer.^132,133^ IOWH-032 is a CFTR inhibitor that also suppresses SARS-CoV-2 replication^134^ and GS-6620 is a precursor of the antiviral drug remdesivir and assessed as treatment against the Ebola virus.^135^ These compounds might be tested in future experimental studies to extend the available data for QSPR modeling and establish pOx/pOzi-based DDS for more therapeutic fields.

### Prediction Tool

**Figure 10:**
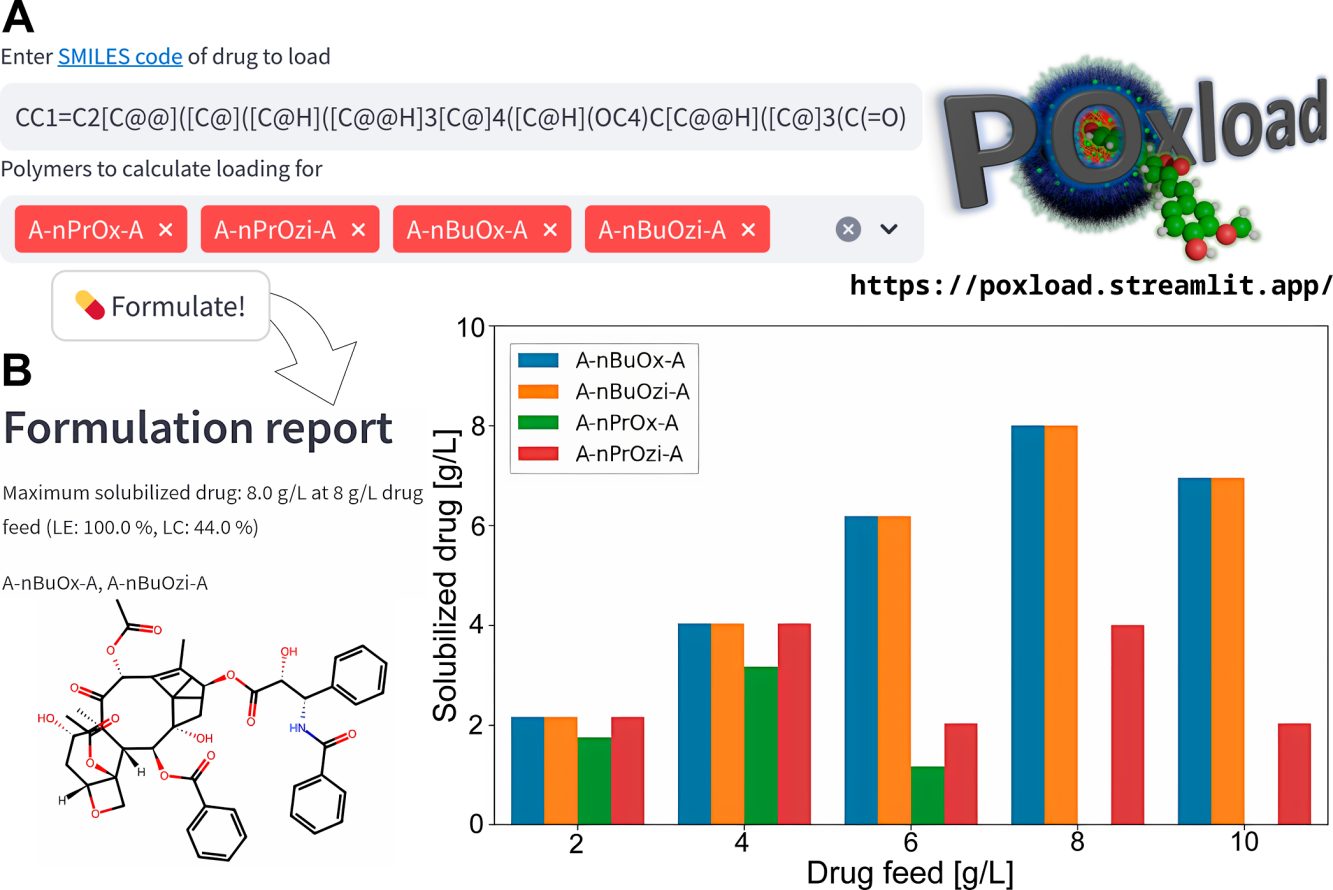
(A) Excerpt from the interface of the web tool POxload, currently accessible at https://poxload.streamlit.app/. (B) Example formulation report output for paclitaxel, providing solubilization estimates for different polymers and DF (average by LE and LC models).

In order to assist other researchers experiencing solubility issues for drugs or potential novel drug candidates, the presented models are integrated into a web application called POxload (https://poxload.streamlit.app/). In the current version, users can predict LC and LE values for single molecules or coformulations by providing corresponding SMILES strings (Figure 10A). Different polymers can be selected for which drug loading is predicted, with the most common variants (A-nPrOx-A, A-nPrOzi-A, A-nBuOx-A, and A-nBuOzi-A) listed as default. Predictions can be made either by the final models (Table 1) or by the best subset/model type combination (RDK7-RF) that showed good Acc values as well (Table S3). From the thresholds passed, the maximum of the solubilized drug is computed (*e.g.*, LE = 39% when passing LE20 but not LE40), given a polymer feed of 10 g/L and DF from 2 to 10 g/L. Predictions are listed for both parameters individually and in a combined way, listing the average amount of solubilized drug predicted by both LC and LE models (Figure 10B). For calculation of these values, the application determines the last threshold passed before two consecutive thresholds result in negative predictions. However, in general, we recommend users to evaluate the predictions of all LC and LE models for deciding whether to investigate the selected drug experimentally; as discussed above, LC models are more likely to result in positive predictions at higher DF, whereas high LE values are more common at lower DF values. For predictions outside of the AD, values for a negative prediction are assumed for calculation of the solubilized drug. In the end of each formulation report, predictions for all individual models are also shown as barplots and listed in a tabulated format. The tool also includes a batch mode and the option to evaluate long-term storage for single polymers. The software is currently hosted and downloadable as a command-line tool at our github page (https://github.com/juppifluppi/poxload).

## Conclusions and Outlook

In this work, we collected the experimental data of several recent publications to create an openly accessible, extended formulation database of drug-loaded pOx- and pOzi-based amphiphilic micelles (Table S1). These data were harnessed to build classification models with different thresholds for LC and LE that, overall, achieved comparable statistics to models previously built on a subset of the data,^35^ with Acc values around 0.8 for CV and subsequent external HS predictions. Instead of SiRMS descriptors, the final selection of models uses molar fraction-weighted mordred descriptors and RDK fingerprints for conveying information about physicochemical properties and important structural elements. While we are not aware of another QSPR study revolving around block copolymers for pharmaceutical applications beyond the SiRMS-based approaches of Alves *et al.*(35) and Rakhimbekova *et al.*,^36^ our additional ways of mixture descriptor generation can be most closely compared to the work of Rasulev *et al.*(32) that successfully modeled the applicability of polymer coating materials for prevention of marine biofouling. In a similar fashion to what was performed in the present study, they modeled complex, multicomponent polymer coatings by weighting the properties of individual repeating units by their concentrations within the mixture. Our results indicate that such a methodology can be applied to other polymer modeling tasks as well.

Compared to the previous work of Alves *et al.*,^35^ the data set was extended significantly in terms of long-term stability measurements and information on a large variety of polymer compositions. Performing more measurements, especially in terms of diversifying the selection of drugs for which loading is investigated with multiple polymer compositions under various conditions, could further improve both the modelability of the data set and our understanding of the effects of tuning the experimental settings. Furthermore, as recently demonstrated by Rakhimbekova *et al.*,^36^ if new compounds are selected with the models for experimental screening, active learning approaches on relatively small sets of initial measurements could further optimize the polymer selection process.

The importance of features of our models investigated *via* SHAP analysis suggests that a hydrophobic cargo is suited for loading with these micelles if it contains a sufficient amount of polar elements in the form of, *e.g.*, hydroxyl groups, ethers, or substituted rings, presumably due to interactions with the amide moieties of the polymeric delivery system. These may be established directly through hydrogen bond donor groups or, more indirectly, through bridging water molecules, as it has been shown that these polymeric micelles contain significant amounts of water.^45,46,48^ Furthermore, the molecular topology of each micellar constituent plays an important role in the model outcome and leads to successful discrimination of favorable polymers for loading of paclitaxel and curcumin.

A virtual screening approach for compounds of the DrugBank demonstrated the high-throughput applicability of our models and suggested additional drugs for future formulations. Given an ensemble of models with multiple thresholds, the tool POxload will be used as a complementary *in silico* tool alongside our own research to rapidly provide a first assessment of the drug loading properties of potential new formulations and thus potentially narrow down the necessary experimental workload. As such, the herein presented tool can be seen as an easily applicable prefiltering method for the detection of drugs that could be efficiently formulated with already established pOx/pOzi-based delivery systems, prior to time-intensive experimental measurements or large-scale molecular dynamics simulations^48^ that could be necessary for dissecting the optimal drug-polymer combination more precisely. Vice-versa to predicting the loading of a drug with known polymers as vehicles, we envision that collection of large amounts of additional data—through experiments, data mining approaches,^136^ derivation of simulation-based formulation fingerprints,^137,138^ and extension of our method to more polymers—could eventually allow us to establish a prediction model for designing suitable polymeric vehicle systems for any kind of poorly soluble compound, made possible through the great tunability of pOxi/pOzi sidechains. The potential of designing optimized polymers in such a way with the help of QSPR models was previously already demonstrated; macromolecules with increased refractive indices could successfully be found using virtual libraries generated through derivatization of promising starting elements.^27^

## Supporting Information Available

Y-randomization results; regression models; SHAP analysis of final models; bivariate scatterplots of LC values and hydrogen bond forming functional groups; additional ALE plots; and further details on relevant molecular descriptors (PDF)
Formulation database, including SMILES codes and naming schemes for polymers, monomers, drugs, and solvents; validation metrics for all regression models; validation metrics for all classification models; virtual screening results of the DrugBank for the final models; and screening results for the RDK7-RF subset models (XLSX)
