# Supplementary material for: POxload: Machine Learning Estimates Drug Loadings of Polymeric Micelles
Source: Mol Pharm. 2024 May 28;21(7):3356–74. doi: 10.1021/acs.molpharmaceut.4c00086 (PMC11394009; doi:10.1021/acs.molpharmaceut.4c00086)
Supplement: Supplementary file 1 — mp4c00086_si_001.pdf [file mp4c00086_si_001.pdf]

Supporting Information
POxload: Machine Learning Estimates Drug Loadings of Polymeric Micelles

Josef Kehrein,[*,†,‡] Alex Bunker,[‡] and Robert Luxenhofer[†]

†*Soft Matter Chemistry, Department of Chemistry, Faculty of Science, University of Helsinki, A. I. Virtasen aukio 1, 00014 Helsinki, Finland*

‡*Drug Research Program, Division of Pharmaceutical Biosciences, Faculty of Pharmacy, University of Helsinki, Viikinkaari 5 E, 00014 Helsinki, Finland*

* Email: josef.kehrein@helsinki.fi

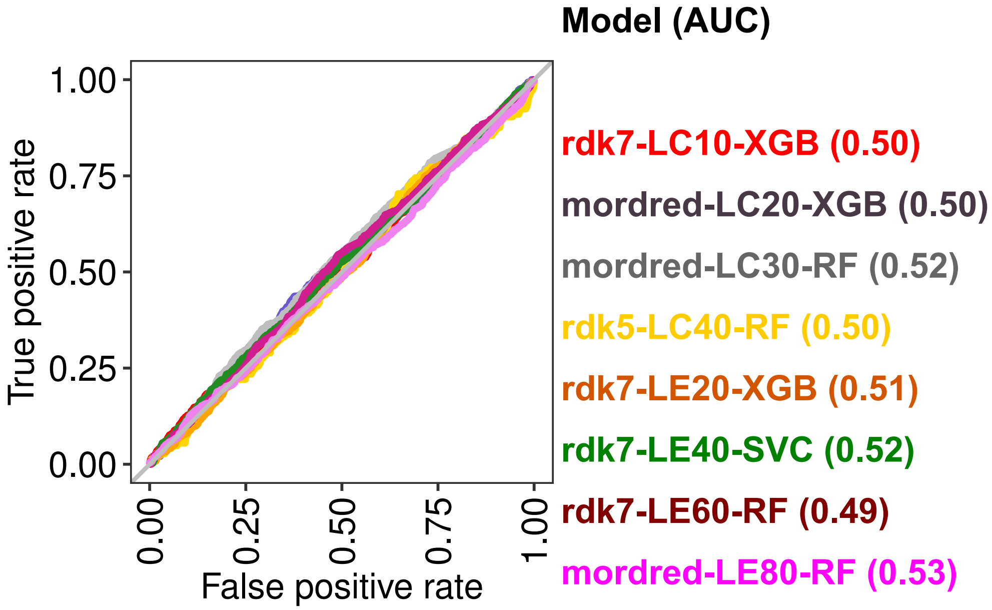

Figure S1: AUC curves for the final models (see Table 1) from a Y-randomization modeling process, suggesting no chance-correlation.

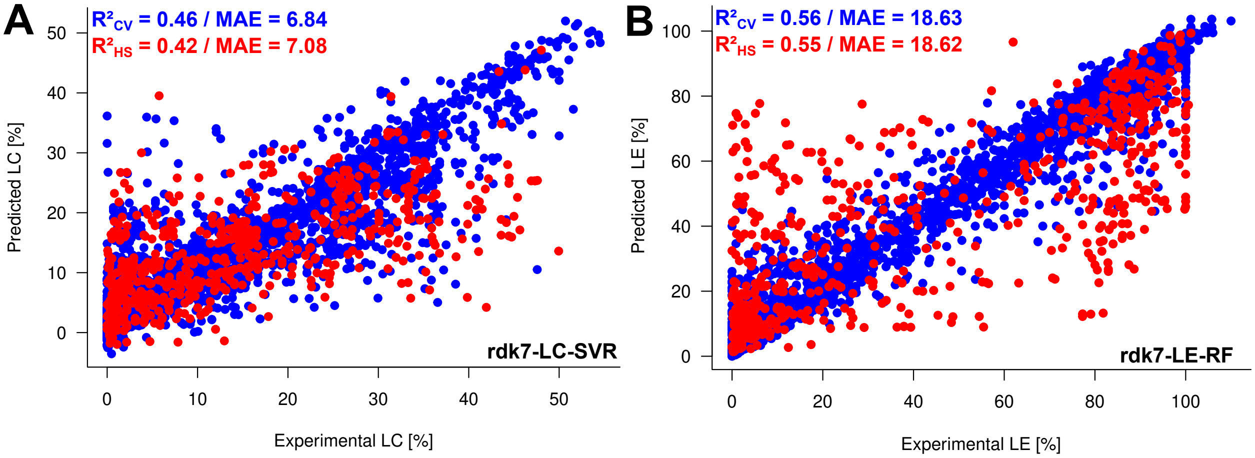

Figure S2: Plots for TS and HS predictions of the best regression models for LC and LE values (rdk7-LC-SVR and rdk7-LE-RF). On the top left mean values of the cross-validated R^2_{CV} scores and MAE_{CV} values and corresponding HS statistics are reported (see also Table S2).

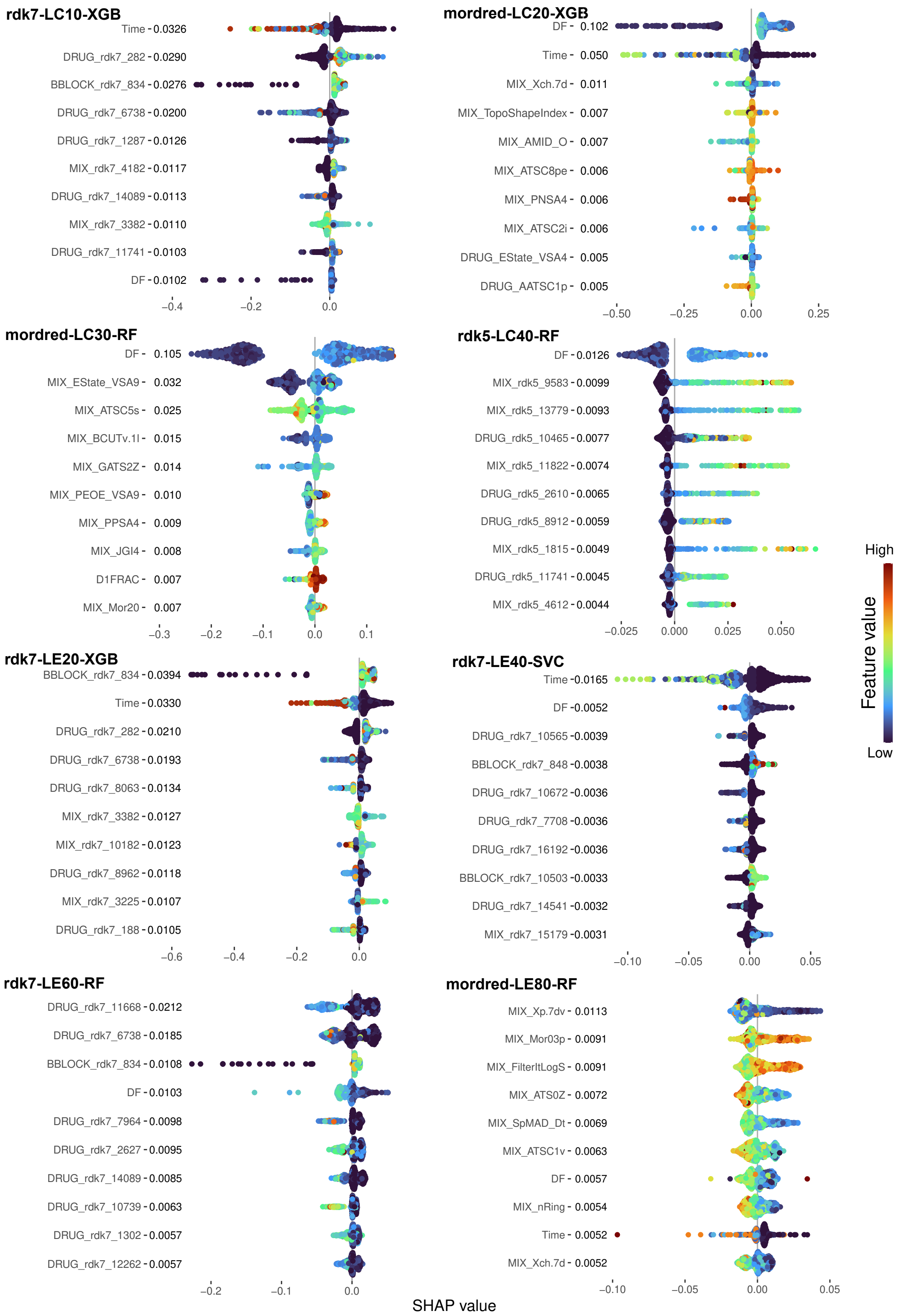

Figure S3: Top 10 features of each model from Table 1, sorted according to mean absolute SHAP values (listed next to each descriptor name). Each data point is colored according to the respective descriptor value. Negative SHAP values correspond to formulations where the corresponding feature supports a negative prediction (threshold not passed), whereas points where the descriptor value contributes to a positive model prediction (threshold passed) are assigned larger SHAP values.

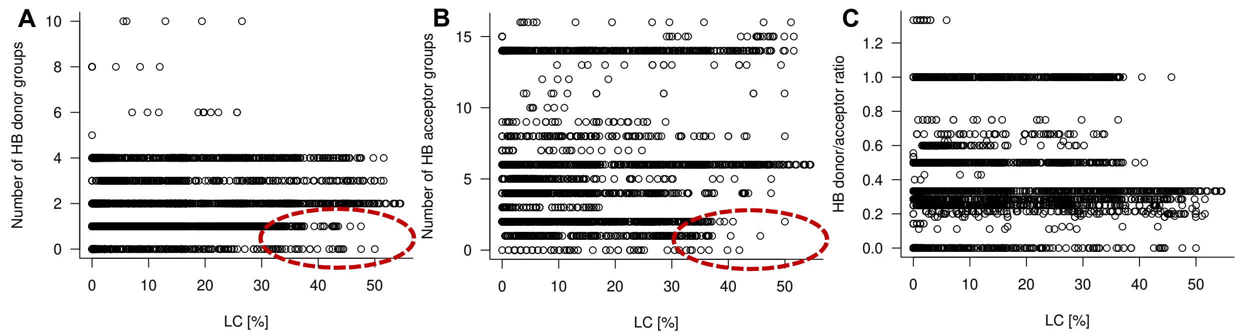

Figure S4: Bivariate scatter plots for LC values of the whole formulation database (TS + HS) and (A) the number of drug hydrogen bond (HB) donors, (B) acceptors and (C) the corresponding ratio. Red markings illustrate that drugs with high LC values above 30 % contain at least small amounts of HB functional groups for most cases.

Molecular Desciptors

Further information on molecular descriptors can be obtained from the mordred documentation.[1] In the following, connectivity indices and autocorrelation descriptors are shortly described. Chi connectivity indices are computed via the following formula:

$$\chi^m_{ch/p} = \sum_{i=1}^{A} c_i^m = \sum_{i=1}^{A} \left(\prod_{k=1}^{m+1} (p_k - h_k)^{-\frac{1}{2}} \right)$$

where m corresponds to the respective bond order (e.g. 7 for $Xp - 7dv$, encompassing $m + 1$ atoms) for which fragments (ch = chains or p = paths) within the molecule are counted, taking into account all A heavy atoms. For each fragment, c_i^m is computed, where p_k represents the number of sigma or valence electrons and h_k the number of bonded hydrogens of each atom k within the corresponding subgraph.[2]

Moreau-Broto autocorrelation descriptors are computed the following way:

$$ATSXY = \sum_{i=1}^{A} \sum_{j=1}^{A} \delta_{ij} w_i w_j$$

where w_i and w_j are some normalized atomic properties Y of atoms i and j (e.g. the van der Waals volume) that are X atoms apart, and δ_{ij} is the Kroneker delta.[3,4]

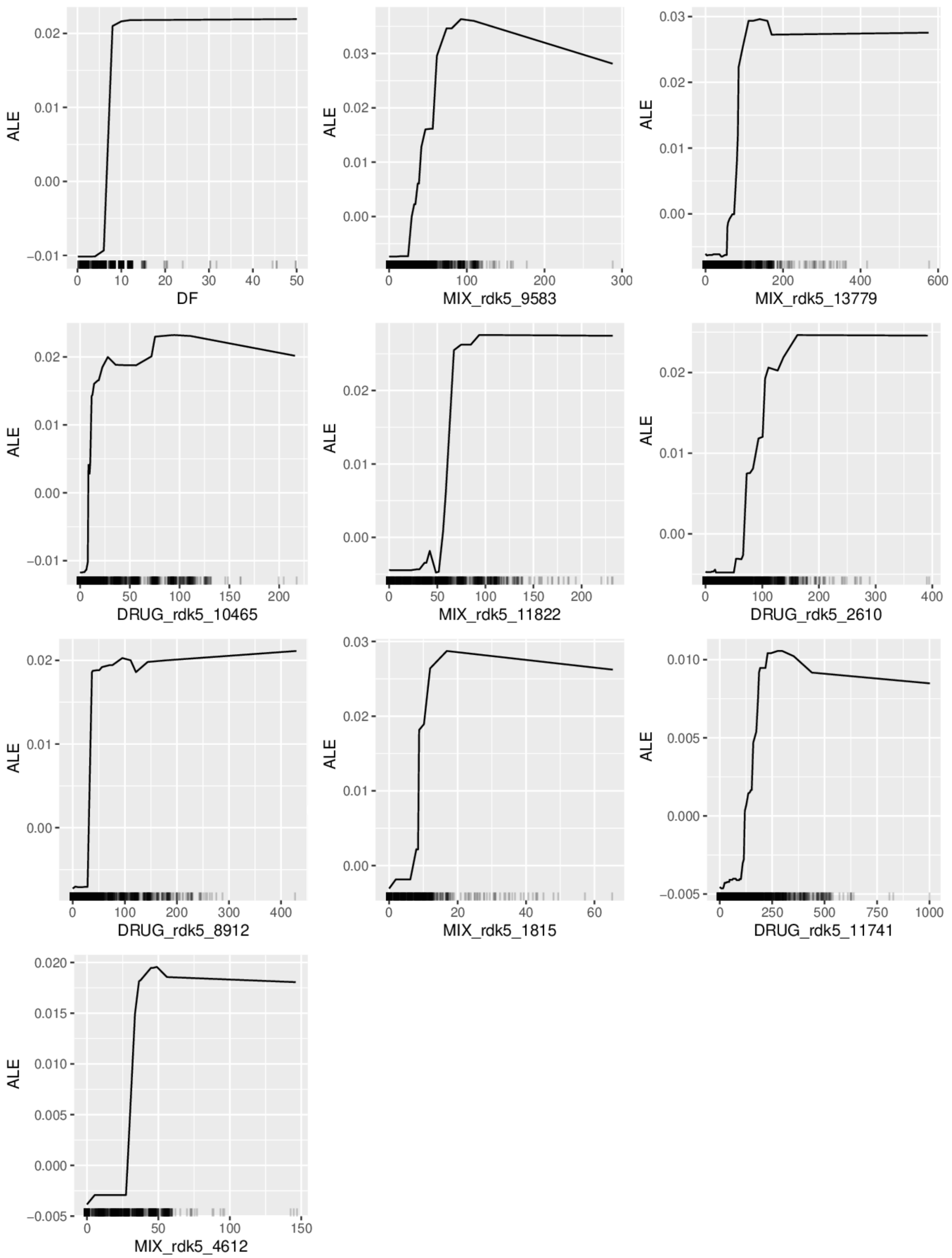

Figure S5: ALE values for the top 10 SHAP features of model rdk5-LC40-RF.

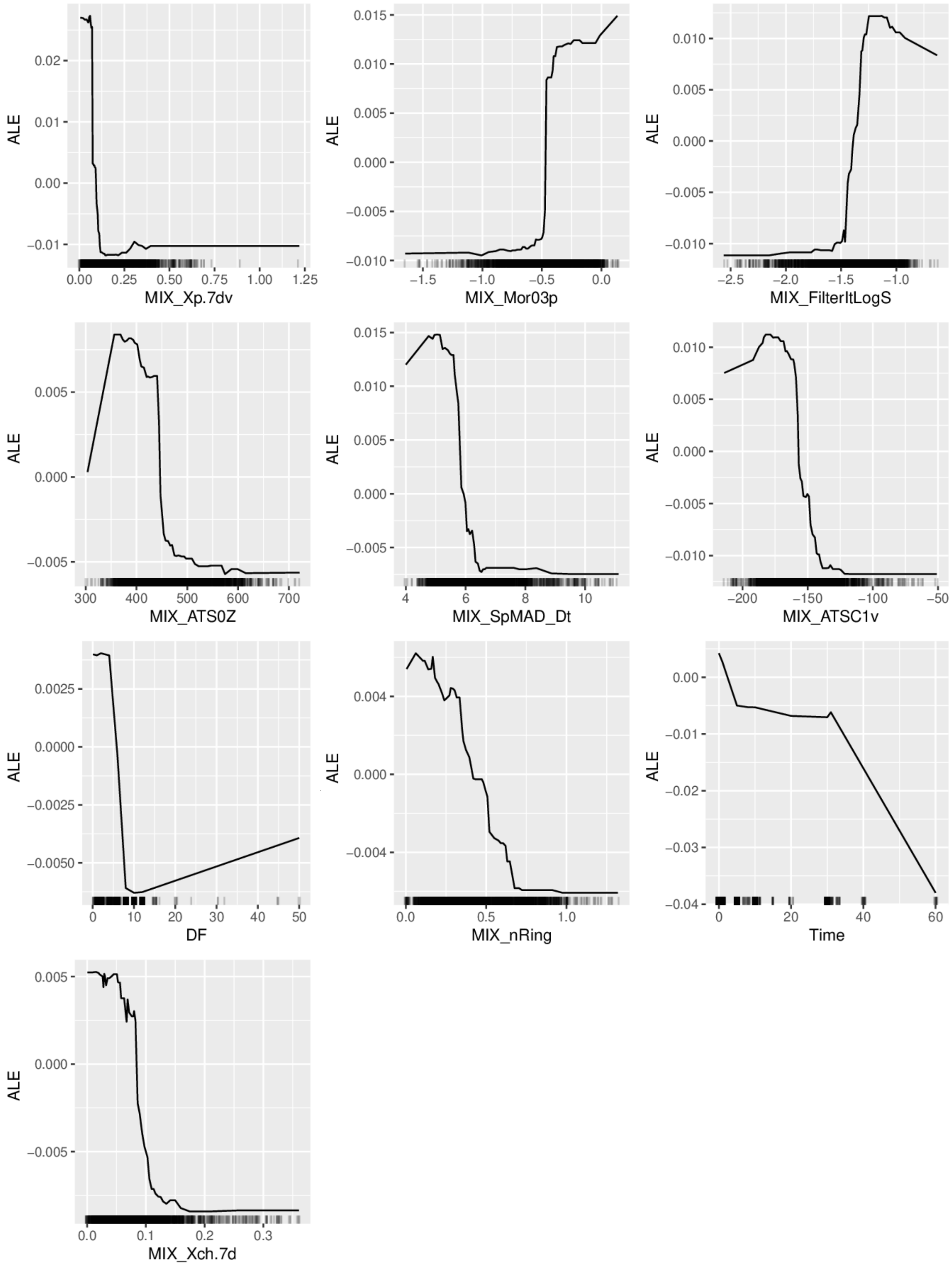

Figure S6: ALE values for the top 10 SHAP features of model mordred-LE80-RF.

References

(1) Moriwaki, H.; Tian, Y.-S.; Kawashita, N.; Takagi, T. Mordred: A Molecular Descriptor Calculator. *Journal of Cheminformatics* **2018**, *10*, 4.

(2) Hall, L. H.; Kier, L. B. In *Reviews in Computational Chemistry*; Lipkowitz, K. B., Boyd, D. B., Eds.; John Wiley & Sons, Inc.: Hoboken, NJ, USA, 2007; pp 367–422.

(3) Broto, P.; Moreau, G.; Vandycke, C. Molecular Structures: Perception, Autocorrelation Descriptor and Sar Studies. Perception of Molecules: Topological Structure and 3-Dimensional Structure. *Eur. J. Med. Chem.* **1984**, *19*, 61–65.

(4) Engel, T.; Gasteiger, J. *Chemoinformatics: Basic Concepts and Methods*; Wiley: Weinheim, Germany, 2018.
